# Prevalence of Intoxicating Substance Use Before or During Sex Among Young Adults: A Systematic Review and Meta-Analysis

**DOI:** 10.1007/s10508-023-02572-z

**Published:** 2023-03-10

**Authors:** María Isabel Gómez-Núñez, Cristian Molla-Esparza, Natalia Gandia Carbonell, Laura Badenes Ribera

**Affiliations:** 1 Department of Research Methods and Diagnostics in Education, International University of La Rioja, UNIR, Logroño, La Rioja Spain; 2 Department of Research Methods and Diagnostics in Education, University of Valencia, UVEG, Av. de Blasco Ibáñez, 30, 46010 Valencia, Spain; 3 Llaurant la Llum Therapeutic Community, Center for the Treatment, Withdrawal and Detoxification of Addictions and Other Associated Disorders, Valencia, Spain; 4 Department of Psychobiology and Health Sciences Methodology, Autonomous University of Barcelona, UAB, Bellaterra, Spain; 5 Department of Methodology of the Behavioural Sciences, University of Valencia, UVEG, Valencia, Spain

**Keywords:** Sexual risk behavior, Sex, Drugs, Young adults, Meta-analysis, Substance use

## Abstract

Drug use before or during sex is a high-risk sexual behavior associated with adverse health risks and outcomes, such as increasing the likelihood of overdoses and of acquiring sexually-transmitted diseases. This systematic review and meta-analysis of three scientific databases examined the prevalence of the use of intoxicating substances, those tending to excite or stupefy the user on a psychoactive level, before or during sex, among young adults (18–29 years old). A total of 55 unique empirical studies met the inclusion criteria (48,145 individuals; 39% males), were assessed for risk of bias using the tools of Hoy et al. (2012), and were analyzed via a generalized linear mixed-effects model. The results produced a global mean prevalence of this sexual risk behavior of 36.98% (95% CI: 28.28%, 46.63%). Nonetheless, significant differences were identified between different intoxicating substances, with the use of alcohol (35.10%; 95% CI: 27.68%, 43.31%), marijuana (27.80%; 95% CI: 18.24%, 39.92%), and ecstasy (20.90%; 95% CI: 14.34%, 29.45%) significantly more prevalent than that of cocaine (4.32%; 95% CI: 3.64%, 5.11%), heroin (.67%; 95% CI: .09%, 4.65%), methamphetamine (7.10%; 95% CI: 4.57%, 10.88%), and GHB (6.55%; 95% CI: 4.21%, 10.05%). Moderator analyses showed that the prevalence of alcohol use before or during sex differed according to geographical sample origin, and increased as the proportion of ethnic whites in samples increased. The remaining demographic (e.g., gender, age, reference population), sexual (e.g., sexual orientation, sexual activity), health (e.g., drug consumption, STI/STD status), methodological (e.g., sampling technique), and measurement (e.g., timeframe) variables that were examined did not moderate prevalence estimates. Implications for sexual development interventions were discussed.

**Supplementary Information:**

The online version contains supplementary material available at 10.1007/s10508-023-02572-z.

## Introduction

Young adulthood is an intensive developmental period often characterized by novel sexual experiences and sensation seeking, which may lead to increased engagement in sexual risk behaviors with the potential to compromise health (Arnett, 2014; Vail-Smith et al., 2010). The use of intoxicating substances before or during sex, which may facilitate sexual contact between youths, is one such experience that has historically been studied (Marcantonio & Jozkowsky, 2021; Weatherburn et al., 2017).

While the use of alcohol or other intoxicating substances, which tend to excite or stupefy the user on a psychoactive level, before or during sex is certainly nothing new, over the last few years several new terms have emerged from academic literature to describe this behavior, such as “sexualized drug use” (hereinafter also referred to as SDU) (Edmundson et al., 2018; González-Baeza et al., 2018). Other terms refer more specifically to the specific substances used and way of administering them (Race et al., 2021). For example, the before and during sex use of substances such as mephedrone, crystal methamphetamine, alkyl nitrites (or poppers), gamma hydroxybutyrate (GHB), and gamma-butyrolactone (GBL), which are mainly orally or nasally ingested, has been labeled as “chemsex” (Drevin et al., 2021). The literature has also referred to chemsex, to a lesser extent, using terms such as “chemsex partying”, “Party and Play” (PnP, P&P), “High and Horny” (HnH, H&H), “intensive sex parties”, and “wired play” (Drysdale, 2021; Hurley & Prestage, 2007, 2009; Meléndez-Torres & Bourne, 2016; Race, 2015). Sexualized drug use via intravenous injection, on the other hand, has been dubbed “slamsex” (Bourne et al., 2015b).

The main motivation for the use of such intoxicating substances before or during sex is to pursue pleasure, which, in turn, involves other motivations, varying from losing inhibitions and facilitating the sexual setting to improving sexual performance and arousal (Bourne et al., 2015a; Piyaraj et al., 2018; Schmidt et al., 2016). Sexual pleasure seeking and experimentation have come to involve new behavioral patterns concerning the intersection between drug consumption and a variety of sexual practices that transgress normative means of pleasure (e.g., sober forms of sexual pleasure) and that demand greater consideration when promoting safe and healthy sexual relationships (Ford et al., 2021a; Piyaraj et al., 2018). Nonetheless, the most empirical literature seems not to have been overly explicit about specific purposes, favoring the referencing of sexual motivation in general (Guerra et al., 2020). SDU and its derivative terms have been socially constructed, and it is, accordingly, likely that the subset of practices involved varies considerably across countries and over time (Maxwell et al., 2019).

Studies have shown that the consumption of intoxicating substances before or during sexual activity can lead to the loss of inhibitions and an increase in self-confidence and perceived attractiveness in relation to others (Palamar et al., 2018b; Santos et al., 2018). For example, one study on the sexual effects of drugs in a sample of young adults has documented various associated psychophysiological effects, such as greater sexual outgoingness, orgasm intensity, and length of sexual interaction (Palamar et al., 2018b). Though, such effects may not exclusively be due to the use of recreational intoxicating substances, but also to prescription or over-the-counter pharmacological treatments aimed at treating symptoms of a disease or condition (e.g., mental health disorders, impotence), especially in clinical samples. However, to our knowledge, no empirical studies have yet been carried out specifically to assess potential confounding factors of sexual experiences and their psychophysiological effects under the influence of intoxicating substances among young adult populations.

The use of intoxicating substances before or during sex also entails certain adverse health risks and outcomes. A large part of the literature has focused on studying sexual-related consequences, such as demonstrating that pre-sex drug consumption is associated with an early age of sexual debut (Kebede et al., 2018; Tan et al., 2021), having multiple partners (Edmundson et al., 2018; Huibregtse et al., 2021), inconsistent condom use (Ristuccia et al., 2018; Strandberg et al., 2019), or unplanned pregnancies (Dong et al., 2015; Metzger, 2015). Using intoxicating substances before or during sex has also been associated with various negative health outcomes, including overdoses (Hammoud et al., 2017; Hegazi et al., 2017), and a greater likelihood of acquiring blood-borne viruses, such as human immunodeficiency virus (HIV), and other sexually transmitted infections (STIs) (González-Baeza et al., 2018; Mayo-Wilson et al., 2020).

To date, a number of empirical and review studies have examined SDU prevalence and its associated outcomes, predominantly among gay and bisexual communities, and communities of other men who have sex with men (MSM) (Edmundson et al., 2018; Guerra et al., 2020; Íncera-Fernández et al., 2021; Lafortune et al., 2021; Maxwell et al., 2019), despite SDU not being exclusive to these populations. Two reviews aimed at estimating SDU and chemsex prevalence among such populations suggest high and heterogeneous prevalence estimates (Edmunson et al., 2018; Maxwell et al., 2019). One review carried out by Edmunson et al. (2018) found that prevalence estimates of SDU and chemsex ranged from 4 to 41% and 17 to 31%, respectively, among MSM in the United Kingdom. Another review, by Maxwell et al. (2019), contributed to the field with an estimated prevalence range of chemsex-related behavior between 3 and 29%. In this study, prevalence estimates were also found to vary according to the particular substance used: methamphetamines (3–22%), cocaine (2–33%), and ketamine (1–4%).

However, there is a general lack of review studies aimed at the use of intoxicating substances before or during sex among young adults in particular, despite there being an increasing number of empirical studies aimed at estimating its prevalence in this age group, with high resulting rates (Meuwly et al., 2021). For example, analyses of a survey of United States college students showed that 56% of the sample had drunk alcohol or used drugs prior to sexual contact during the year prior to the survey, with the experience more prevalent with increasing age (Powell, 2018). Another study of Portuguese university students found that approximately 33% of the sample had had intercourse under the influence of alcohol or while taking drugs during the last twelve months, showing significant statistical differences by the sex of the participants. Specifically, men were more likely to combine sex with alcohol (21.4% of women, 51.8% of men) and drugs (4.8% of women, 17.7% of men) (Santos et al., 2018). However, the results of a recent prospective cohort study of high school senior students suggest that women may be making increasing use of alcohol, as they increasingly share common behaviors and exposures with men, including alcohol consumption (McKetta et al., 2022). A further study carried out among United States college students documented that approximately a third of students had consumed substances before having sex the most recent time, and that African American women, in particular, had the lowest frequency of participation in this kind of activity (Vail-Smith et al., 2010). This suggests that while direct comparisons cannot be made a priori, high variations among the prevalence rates reported between studies may be explained by demographic, sexual and health factors, as well as measurement and other methodological factors characterizing the available empirical studies (Lafortune et al., 2021).

In summary, a lack of reviews of the use of intoxicating substances before or during sex among the young adult population, and its potential health implications, supports the need to carry out a thorough review aimed at estimating its prevalence. There is also a need to study the heterogeneity among prevalence rates, by considering study-level characteristics in terms of demographic (e.g., gender, age, ethnicity, sample geographical origin, reference population), sexual (e.g., sexual orientation, sexual activity, mean age of sexual debut, number of sexual partners), health (e.g., drug consumption, STI/STD status), methodological (e.g., sampling, administration procedure), and measurement (e.g., purpose, willingness, substance types, timeframe) variables. Therefore, the main aim of this study is to provide a meta-analytic synthesis of the prevalence of the use of intoxicating substances before or during sex among the young adult population, as well as examine potential moderators that may explain the observed heterogeneity in prevalence rates. Due to the disparities among individual empirical studies and the lack of reviews concerning young adults, this research is of an exploratory nature, and, therefore did not contemplate the formulation of hypotheses. Ultimately, knowing the extent of the use of intoxicating substances before or during sex among the young adult population will facilitate the planning of preventive sexual health strategies and interventions for this specific age grouping.

## Method

### Study Design

The systematic review and meta-analysis were conducted according to an established protocol registered on the International Platform of Registered Systematic Review and Meta-Analysis Protocols (INPLASY No. 2021100077), following the Preferred Reporting Items for Systematic Reviews and Meta-Analyses Protocols (PRISMA-P) guidelines. In particular, the study followed Appendix 1 and 2 of the Preferred Reporting Items for Systematic Reviews and Meta-Analyses (PRISMA 2020) statement (Moher et al., 2009), and the Guidelines for Reporting Systematic Reviews and Meta-Analyses (Rubio-Aparicio et al., 2018).

### Search Strategy

The study search was carried out between July and October 2021, mainly using three electronic databases: ISI Web of Science (WoS Core Collection) via Thomson Reuters; Scopus, via Elsevier; and Psychological Information (PsycINFO), via APA PsycNET. Following the Peer Review of Electronic Research Strategies guideline (McGowan et al., 2016), the search strategy was built through an iterative process (empirical literature key terms, and database consultations), which also considered the aforementioned non-age-specific reviews. The strategy included both highly sensitive and unspecific, comprehensive search descriptors and expressions referring to both the use of intoxicating substances before or during sex and young adults as the target population (Supplementary Tables S1a–S1d). The key words were combined in different ways and the search strategy was modified according to the specific requirements of each database. The search was limited to the timeframe from the year 2000–2021. The references of relevant published studies were also examined in order to acquire potentially eligible documents consistent with our search criteria. Furthermore, grey literature was searched via Google and Google Scholar to obtain other potentially eligible studies, such as reports, theses, unpublished papers, government documents, and so on. Finally, emails were sent to the principal researcher of research groups that had published the most about the use of intoxicating substances before or during sex involving young adults, with the aim of identifying hitherto unpublished studies that might not have been obtained by the other means.

### Study Selection Criteria

Studies were included if they: (1) reported a prevalence figure of drug use before or during sex, including any prevalence disaggregated by substance or participant sex; (2) provided original empirical data; (3) comprised a sample of participants from 18 to 29 years old; and (4) were published or available in English, Spanish or Catalan. Studies were excluded if they: (1) provided insufficient demographic or methodological descriptions; (2) reported a prevalence not calculable from the data; or (3) considered the same sample and reported the same results as another of the studies already included. Furthermore, clinical and community specific samples (e.g., injected drug users), which might bias the prevalence results, were excluded. Study titles and abstracts and full-text inclusion criteria were screened independently by two researchers. Disagreements were resolved through discussion, and, if necessary, a third researcher was consulted.

### Study Coding

The coding process was conducted independently and duplicated by another two researchers using a standard data extraction form. Once again, any discrepancies were resolved by consensus, and a third researcher was consulted if required. Effect size data included the proportion of participants that had used intoxicating substances before or during sex, whether reporting a global prevalence (i.e., any drug) or multiple prevalences disaggregated by substance type. In certain studies, additional calculations were required to determine prevalence. From longitudinal study designs, only the baseline data were considered.

In addition, the following relevant bibliometric, demographic, sexual, health, methodological, and measurement information was extracted: (1) document type (e.g., article, degree, master or doctoral thesis, report, peer-reviewed or not, under review); (2) publication year; (3) data collection year; (4) sample geographical origin; (5) study design (e.g., cross-sectional, longitudinal); (6) sampling technique (e.g., probabilistic, non-probabilistic); (7) reference population (e.g., university students; men who have sex with men); (8) sample size; (9) participant gender distribution; (10) participant sexual orientation distribution (i.e., proportion of heterosexuals); (11) participant ethnicity distribution (i.e., proportion of whites); (12) socioeconomic status; (13) range, mean and standard deviation of the age of participants; (14) administration procedure (e.g., online survey, face-to-face interviews); (15) sexual practice related aspects (e.g., proportion of participants sexually active, mean age of sexual debut, mean of sexual partners); (16) drug consumption (e.g., proportion of consumers of specific intoxicating substances); (17) relationship context of participants using drugs before or during sex (e.g., romantic, casual); (18) timeframe (e.g., last sex, lifetime); (19) purpose of using drugs before or during sex (e.g., to enhance the sexual experience); (20) willingness to partake in the use of intoxicating substances before or during sex (e.g., voluntary, solicited); and (21) sexually transmitted disease status.

### Study Quality

The risk of study bias was assessed using an ad-hoc tool elaborated by the authors and adapted from a tool specifically designed to assess bias in prevalence studies (Hoy et al., 2012). This tool evaluates five methodological quality domains, three relating to internal validity, and two relating to external validity: (1) common administration procedures across all participants (yes or no); (2) measure quality (reported or justified evidence of validity and reliability in the study sample, or equivalent samples); (3) timeframe (well-defined or undefined); (4) representativeness of the target population (probabilistic or non-probabilistic sample); and (5) study response rate. The potential moderating effect of study quality on prevalence estimates was then assessed and is discussed in the results section.

### Statistical Analysis

The meta-analysis was conducted using generalized linear mixed models (GLMMs) with a logit link function to estimate the prevalence of intoxicating substance use before or during sex. Previous preliminary meta-analytical studies have suggested that the use of GLMMs to estimate non-normally distributed outcomes, as in the case of proportions, show smaller biases and mean squared errors and higher coverage probabilities than two-step methods (Lin & Chu, 2020). In this study, prevalence estimates are presented with 95% confidence intervals (CIs) and credibility intervals (CRs), and all parameters were estimated using the maximum-likelihood approach. Between-study heterogeneity was analyzed using the Higgins’ inconsistency index (*I*^2^) and tested using the likelihood ratio test. Univariate GLMMs were used to explore possible sources of heterogeneity, including potential categorical (e.g., sampling technique, risk of bias) and quantitative (e.g., data collection year, mean age) moderators as covariates. Classical recommendations were used to assess categorical moderators with at least three studies at each level, and continuous moderators with at least ten studies per covariate (Borenstein et al., 2009). Funnel plot symmetry was analyzed to examine potential publication bias, and forest plots were used to visualize the meta-analysis study results. A *p*-value < .05 was considered statistically significant. The meta-analysis was performed using the *metafor* package in R (version 3.8-1).

## Results

### Search Results and Study Characteristics

**Fig. 1:**
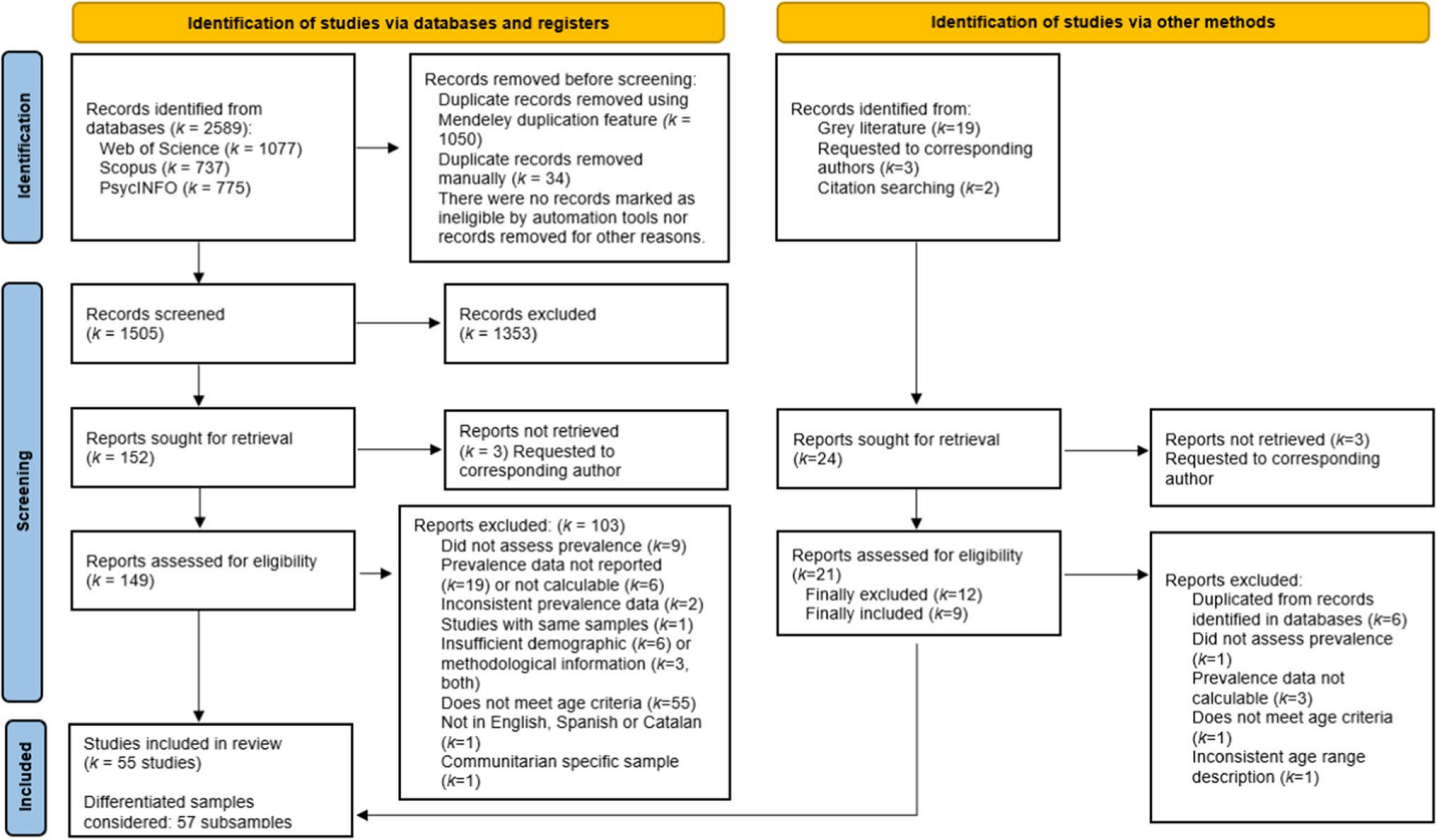
PRISMA flowchart

A total of 2589 documents were identified via the aforementioned databases. After duplicates had been eliminated, 1505 studies were screened on the basis of their title and abstract. Subsequently, 149 potentially eligible reports were examined on the basis of their full text including references (see Supplementary Table S2 for exclusions). After a comprehensive analysis, 46 studies from the databases and 9 studies from other sources that met the five inclusion criteria were considered to form the basis of this meta-analysis (see Fig. 1). Of the 55 studies selected, a total of 57 analytical samples were included. The studies included in the meta-analysis are listed in Appendix 3.

The documents thus analyzed were, mainly, articles published in scientific journals and subject to the peer-review process (*k* = 49, 89.09%). The remaining documents were doctoral theses (*k* = 6, 10.91%). Most of the selected studies were published in the time interval corresponding to the years 2011 to 2021 (*k* = 38, 69.09%). More than half of the selected studies were conducted in the United States (*k* = 36, 64.45%). The most commonly used tools to assess the use of drugs before or during sex were questionnaires administered online or on paper (*k* = 46*,* 83.64%), followed by face-to-face interviews (*k* = 7*,* 12.73%), and mixed administration procedures (*k* = 2*,* 3.64%). Of the 57 analytical samples, 33 reported on university students, whereas 13 reported on general community samples. The remaining samples were of men who have sex with men (5 samples), migrant workers (3 samples) and clinical samples (3 samples). The analytical samples involved a total of 48,145 participants (39% males). Twenty-one studies reported prevalence rates of using intoxicating substances before or during sex without referring to specific drugs (*k* = 21, 38.18%), while thirty-four studies provided a prevalence specifying at least one substance (*k* = 34, 61.82%), of which alcohol (*k* = 33, 60%) and marijuana (*k* = 6, 10.91%) were the most reported, followed by heroin, cocaine, poppers, methamphetamine, and ecstasy (*k* = 3, 5.45%), GHB (*k* = 2, 3.64%), and crack, sedatives, hallucinogens, lysergic acid diethylamide (LSD), ketamine, and speed (*k* = 1, 1.82%). Only five studies reported disaggregated prevalence of two or more individual substances. Detailed information on the studies included is provided in Supplementary Table S3.

### Study Quality

The findings explained below are shown schematically in Table 1. The quality assessment revealed that almost all the studies analyzed were cross-sectional studies (*k* = 45, 81.82%) and used non-probabilistic sampling techniques (*k* = 44, 80%). More than three quarters of the study samples included a complete description of the age of the participants involved (*k* = 44, 80%). Regarding the measure quality, only fourteen of the included studies reported or justified validity and/or reliability of the measure in relation to the study sample or comparable samples (*k* = 14, 25.45%). The predominant timeframes in the included studies were in one’s lifetime or non-defined (*k* = 17, 30.91%), followed by in the last three months (*k* = 11, 20%), and last year (*k* = 9, 16.36%). Almost all of the studies included a common administration procedure for all participants (*k* = 53, 96%). Most studies (*k* = 39, 70.91%) provided no information on the response rate. Among those that reported response rates, these ranged between 63 and 88.50% (in the interquartile range). Lastly, regarding risk of bias, a minority of the studies were assessed as having a low risk of bias (*k* = 3, 5.45%), followed by studies with a moderate risk (*k* = 18, 32.73%), and a high risk (*k* = 34, 61.82%). Supplementary Table S4 shows the detailed critical appraisal of the studies.

**Table 1:** Summary of the critical appraisal of the studies included in the review

Methodological quality domains		Studies (*k* = 55) *k* (%)
Study design	Cross-sectional	45 (81.82%)
	Cohort study	3 (5.45%)
Age description	Full age description	44 (80%)
	Not completely	11 (20%)
Sampling technique	Probabilistic	11 (20%)
	Non-probabilistic	44 (80%)
Measurement quality	Reported or justified validity/reliability	14 (25.45%)
	Not reported or unclear	41 (74.55%)
Timeframe	Well defined	38 (69.09%)
	Lifetime or non-defined	17 (30.91%)
Common administration procedure	Yes	53 (96.36%)
	No	2 (3.63%)
Response rate	Reported	16 (29.09%) IQR: 63–88.50%
Risk of bias	Low	3 (5.45%)
	Moderate	18 (32.73%)
	High	34 (61.82%)

“k” = number of studies, “IQR” = Interquartile range

### Measures of Drug Use Before or During Sex

The findings explained below are shown schematically in Table 2. The use of substances before or during sex were considered in the majority of the studies via a mono-item (*k* = 36, 65.45%). The predominant form of response was dichotomous (yes/no) in 54.54% of the studies (*k* = 30), followed by different Likert frequency scales (*k* = 17, 30.91%). A total of 69.09% of the studies (*k* = 38) considered sexual acts in a general way (e.g., sex, sexual encounter, sexual activity). The presence of penetration was specified in 18.18% of the studies (*k* = 10), while the type of sexual act (e.g., anal, vaginal) was specified in only 7.27% of the studies (*k* = 4). Other aspects considered in a minority of the studies were relationship type (e.g., casual partner) (*k* = 4, 7.27%) and a specific circumstance of the act of sex (i.e., unprotected sex) (*k* = 3, 5.45%). The aspect of participants’ willingness in partaking in the use of intoxicating substances before or during sex was not considered in any of the studies included in the sample. Lastly, only one study specified for the purpose of sex in the measure (*k* = 1, 1.82%).

**Table 2:** Analysis of measures

	Number of studies (*k* = 55)
*Number of items used to assess substance use before or during sex*	
1 item (mono-item)	36
2 or more items	16
Not defined	3
*Response type*	
Dichotomous (yes/no)	30
Likert-type scales	17
Open answer	1
Not defined	7
*Sexual act type*	
Generic (e.g., sexual encounter, sex, sexual activity)	38
Referring to penetration (e.g., sexual intercourse)	10
Specific (e.g., anal, vaginal)	4
*Relationship type*	
Defined (e.g., 2 casual partners, 1 primary partner, 1 non-spousal partner)	4
Not defined	51
*Specific circumstance of the sexual act*	
Defined (e.g., unprotected sex)	3
Not defined	52
*Substance use purpose*	
Defined (i.e., for the purpose of sex)	1
Not defined	54
*Willingness*	
Defined	0
Not defined	55

### Meta-Analytical Results

**Fig. 2:**
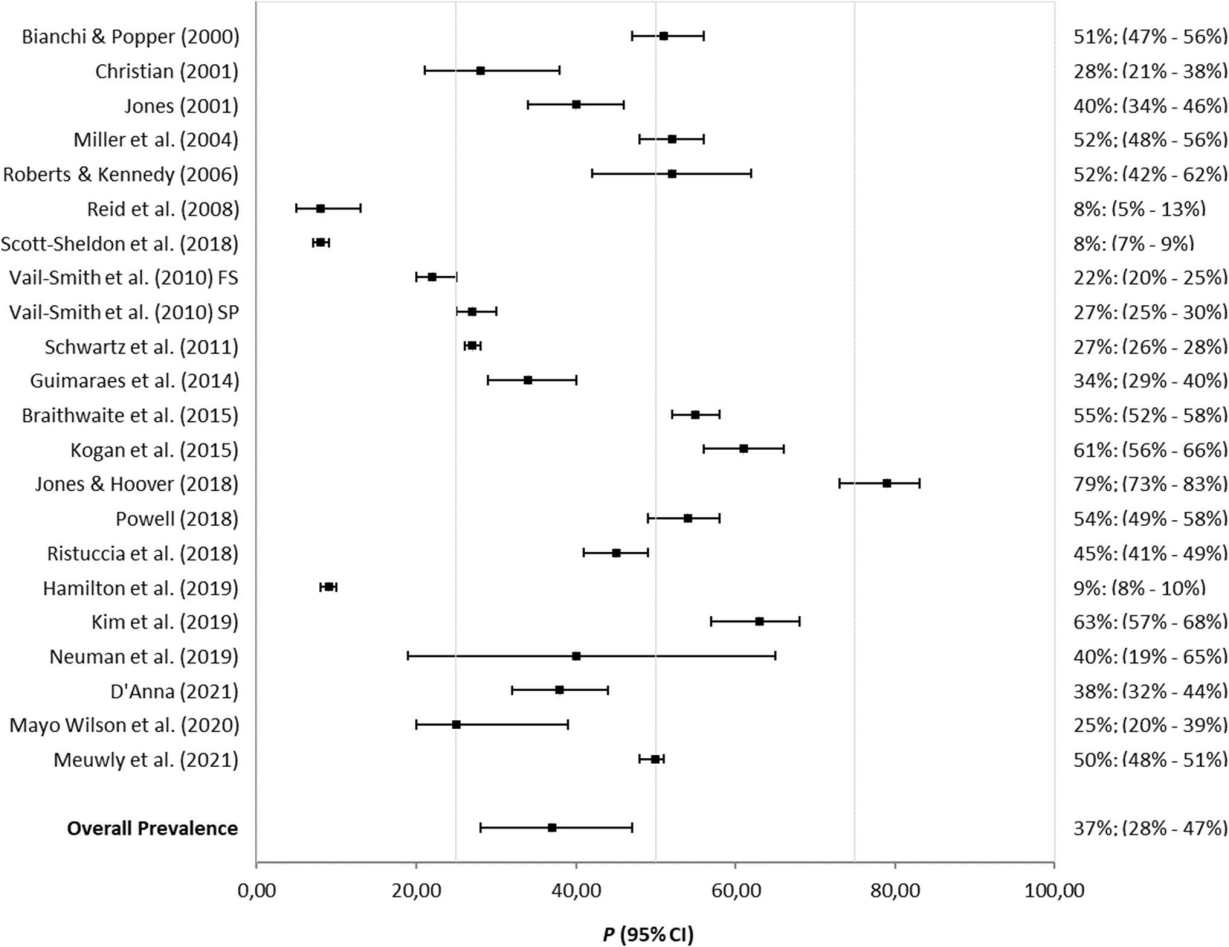
Forest plot of observed prevalences and global mean estimate of intoxicating substance use before or during sex. “95% CI” = 95% Confidence Interval; “FS” = Fall Sample; “SP” = Spring Sample

**Fig. 3:**
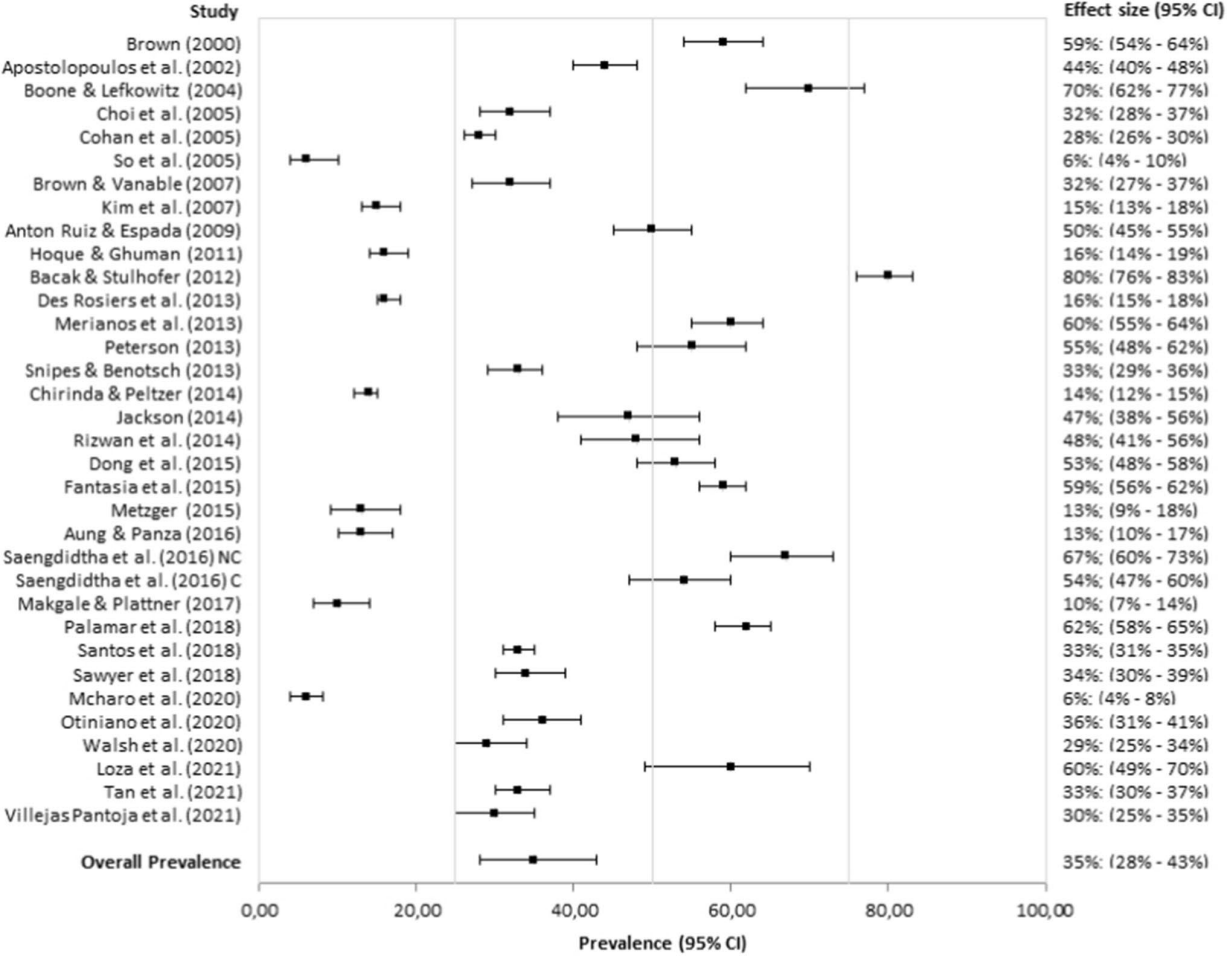
Forest plot of observed prevalences and global mean estimate of alcohol use before or during sex. “95% CI” = 95% Confidence Interval; “NC” = Non-clinical sample; “C” = Clinical sample

The mean prevalence of intoxicating substance use before or during sex without referring to specific substances was 36.98% (95% CI: 28.28%, 46.63%; *I*^*2*^ = 99.40%). Although this is a raw prevalence estimate, it can, however, be used as a proxy for the use of intoxicating substances before or during sex. The observed global prevalence and global mean estimate are illustrated in Fig. 2. As indicated in Table 3, the estimated prevalence of the use of alcohol (35.10%; 95% CI: 27.68%, 43.31%; *I*^*2*^ = 99.12%; see Fig. 3), marijuana (27.80%; 95% CI: 18.24%, 39.92%; *I*^*2*^ = 97.35%), and of ecstasy (20.90%; 95% CI: 14.34%, 29.45%; *I*^*2*^ = 87.60%) before or during sex were significantly higher than the estimated prevalence of the use of cocaine (4.32%; 95% CI: 3.64%, 5.11%; *I*^*2*^ = .46%), of heroin (.67%; 95% CI: .09%, 4.65%; *I*^*2*^ = 86.62%), of methamphetamine (7.10%; 95% CI: 4.57%, 10.88%; *I*^*2*^ = 69.55%), and of GHB (6.55%; 95% CI: 4.21%, 10.05%; *I*^*2*^ = 71.96%), without overlapping confidence intervals. No evidence of publication bias was observed for the global prevalence, or in the case of alcohol use (Appendix 4 and 5). The consumption prevalence of other substances, such as crack, speed, sedatives (or barbiturates), LSD and ketamine, was not meta-estimated since it was reported in only one study.

**Table 3:** Pooled prevalence of intoxicating substance use before or during sex among young adults

	*k*	eff (%)	(95% CI)	(95% CRs)	Tau^2^	*I*^2^	Tests for heterogeneity
Global prevalence (various drugs)	22	36.98	(28.28%, 46.63%)	(8.20%, 79.41%)	.88	99.40%	Wld(df = 21) = 2795.82, *p* < .01 LRT(df = 21) = 3479.11, *p* < .01
Marijuana	6	27.80	(18.24%, 39.92%)	(8.54%, 61.34%)	.45	97.35%	Wld(df = 5) = 237.49, *p* < .01 LRT(df = 5) = 262.19, *p* < .01
Alcohol	34	35.10	(27.68%, 43.31%)	(6.64%, 80.42%)	1.04	99.12%	Wld(df = 33) = 2679.60, *p* < .01 LRT(df = 33) = 3287.62, *p* < .01
Cocaine	3	4.32	(3.64%, 5.11%)	(3.64%, 5.11%)	.01	.46%	Wld(df = 2) = 5.93, *p* = .05 LRT(df = 2) = 4.84, *p* = .09
Heroin	3	.67	(.09%, 4.65%)	(.02%, 17.80%)	2.12	86.62%	Wld(df = 2) = 4.17, *p* = .12 LRT(df = 2) = 13.50, *p* < .01
Methamphetamine	3	7.10	(4.57%, 10.88%)	(3.30%, 14.61%)	.12	69.55%	Wld(df = 2) = 11.51, *p* < .01 LRT(df = 2) = 12.10, *p* < .01
Ecstasy	3	20.90	(14.34%, 29.45%)	(10.32%, 37.76%)	.13	87.60%	Wld(df = 2) = 23.42, *p* < .01 LRT(df = 2) = 24.73, *p* < .01
Poppers	3	8.38	(2.02%, 28.90%)	(.47%, 63.84%)	1.70	98.64%	Wld(df = 2) = 144.33, *p* < .01 LRT(df = 2) = 236.16, *p* < .01
Gamma Hydroxybutyrate	2	6.55	(4.21%, 10.05%)	(3.27%, 12.69%)	.08	71.96%	Wld(df = 1) = 7.07, *p* < .01 LRT(df = 1) = 7.30, *p* < .01

“k” = number of studies included, “eff” = effect size, “95% CI” = 95% Confidence Interval, “95% CRs” = 95% credibility intervals, “Tau^2^” = estimated amount of (residual) heterogeneity, “I^2^” = I-square, “Wld” = Wald-type test statistic of the test for (residual) heterogeneity, “LRT” = Likelihood Ratio Test statistic of the test for (residual) heterogeneity

The prevalence of Crack, Speed, Sedatives/Barbiturates, Lysergic Acid Diethylamide (LSD) and Ketamine was reported by only 1 study

#### Global Prevalence Moderators

The global mean prevalence of intoxicating substance use before or during sex among university students was 33.68% (95% CI: 23.32%, 45.89%), which did not differ from general community samples (47.77%; 95% CI: 32.59%, 63.38%) (*p* = .19) (see Table 4). Estimates for clinical, migrant worker and MSM samples could not be performed because of an insufficient number of studies. As no statistically significant differences were observed by sample type, subsequent analyses were performed on the total number of analytical samples.

**Table 4:** Results of categorical moderator variables on the prevalence of intoxicating substance use before or during sex

	Global Prevalence (various drugs)				Alcohol			
	*k*	eff	(95% CI)	*p*	*k*	eff	(95% CI)	*p*
*Document type*	22			.78	34			.99
Not peer-reviewed	3	40.37%	(18.78%, 66.47%)		3	35.25%	(14.45%, 63.71%)	
Peer-reviewed	19	36.46%	(27.23%, 46.80%)		31	35.08%	(27.35%, 43.69%)	
*Risk of bias*	22							.40
Low risk	0^a^				3	50.24%	(23.35%, 77%)	
High risk	22				19	36.70%	(26.42%, 48.34%)	
*Reference population*				.19				.51
General community	7	47.77%	(32.59%, 63.38%)		6	44.19%	(26.60%, 63.37%)	
University students	11	33.68%	(23.32%, 45.89%)		22	31.64%	(23.49%, 41.10%)	
Men who have sex with men	2				2			
Migrant workers	0				3	35.12%	(15.11%, 62.20%)	
Clinical	2				1			
*Sampling technique*	22				34			.82
Non-probabilistic	21				24	32.99%	(16.69%, 54.75%)	
Probabilistic	1^a^				10	35.47%	(27.44%, 44.41%)	
*Administration procedure*	22				34			.82
Administered by others	2^a^				5	32.99%	(16.69%, 54.75%)	
Self-administered	20				29	35.47%	(27.44%, 44.41%)	
*Timeframe*	22			.93	31			.07
Last sex	5	15.11%	(3%, 50.14%)		2			
During last one to six months	10	32.72%	(22.63%, 44.78%)		9	24.45%	(14.23%, 38.69%)	
During more than last six months	7	39.47%	(31.16%, 48.35%)		20	40.51%	(30.34%, 51.58%)	
*Geographical origin of the sample*^b^	22				34			< .01
Africa	1^a^				4	10.65%	(4.98%, 21.32%)	
Asia	0^a^				6	43.07%	(27.93%, 59.62%)	
Europe	2^a^				3	55.46%	(32.74%, 76.10%)	
United States & Canada	18				21	36.77%	(28.92%, 45.40%)	
South America	1^a^							
*Gender differences*	16			.82				.73
Men	8	44.32%	(33.93%, 55.22%)		5	26.73%	(8.63%, 58.50%)	
Women	8	42.53%	(32.19%, 53.56%)		5	20.77%	(6.34%, 50.38%)	

“k” = number of studies included, “eff” = effect size (mean prevalence), “95% CI” = 95% confidence interval

Analyses examining categorical moderator variables such as purpose, willingness or context in which participants used intoxicating substances before or during sex were not carried out because this information was not specified or was not clearly defined in the primary study measures

Subgroup analysis could not be performed for the substances Marijuana, Cocaine, Heroin, Methamphetamine, Ecstasy, Poppers and Gamma Hydroxybutyrate because of an insufficient number of studies

^a^Insufficient “k” to make comparisons

^b^Prevalence estimates considering the geographical origin of samples were not compared with a significance test, but are provided for descriptive purposes only

As indicated in Table 4, no significant subgroup differences were detected among different categorical moderators concerning the global prevalence of the consumption of intoxicating substances before or during sex (e.g., document type or measure timeframe). It should also be noted that no significant difference was found between men (44.32%; 95% CI: 33.93%, 55.22%) and women (42.53%; 95% CI: 32.19%, 53.56%) (*p* = .82). Certain categorical moderators, including risk of bias, sampling technique, administration procedure, and geographical origin of the sample, could not be analyzed due to an insufficient number of studies. Meta-regression findings (Table 5) showed that the mean prevalence of using intoxicating substances before or during sex significantly decreased in studies with a higher proportion of ethnic whites in their samples (e.g., 65.27%, 95% CI: 41.53%, 83.26%, when the proportion was .15, versus 20.79%, 95% CI: 12.34%, 32.88%, when it was .85, averaging all studies reviewed) (*p* < .01). It was also observed that meta-regression by mean age of sample participants revealed an increasing trend in the use of intoxicating substances before or during sex (e.g., 25.04% at the age of 18, versus 62.99% at the age of 26, averaging all studies reviewed), although the association was not statistically significant. Prevalence was also not moderated by data collection year. Certain quantitative moderators, including the proportion of heterosexuals, of sexually active participants, and the consumption of specific drugs, also could not be analyzed due to an insufficient number of studies.

**Table 5:** Results of quantitative moderator variables on the prevalence of intoxicating substance use before or during sex

	Global Prevalence (various drugs)				Alcohol			
	*k*	Coefficient/eff	(95% CI)	*p*	*k*	Coefficient/eff	(95% CI)	*p*
*Year of data collection*	17	− .00	(− .06, .05)	.87	23	− .02	(− .08, .03)	.39
1999		36.08%	(23.12%, 51.45%)			41.21%	(25.32%, 59.17%)	
2009		35.08%	(25.89%, 45.52%)			35.35%	(26.85%, 44.88%)	
2019		34.09%	(20.03%, 51.65%)			29.90%	(18.12%, 45.12%)	
*Proportion of ethnic whites*	12	− 2.81	(− 4.74, − .88)	< .01	13	1.97	(.70, 3.24)	< .01
.15		65.27%	(41.53%, 83.26%)			23.44%	(14.26%, 36.05%)	
.50		41.26%	(30.75%, 52.64%)			37.93%	(31.34%, 45%)	
.85		20.79%	(12.34%, 32.88%)			54.95%	(44%, 65.45%)	
*Proportion of heterosexuals*	4^a^				13	− .91	(− 5.39, 3.57)	.69
.15						59.22%	(4.65%, 97.74%)	
.50						51.35%	(14.32%, 86.95%)	
.85						43.41%	(32.99%, 54.46%)	
*Mean age of the sample*	18	.20	(− .05, .46)	.11	30	− .01	(− .26. .24)	.91
18		25.04%	(13.38%, 41.93%)			35.46%	(19.18%, 55.98%)	
22		42.99%	(29.64%, 57.44%)			34.20%	(25.21%, 44.49%)	
26		62.99%	(28.48%, 87.91%)			32.96%	(11.86%, 64.25%)	
*Proportion sexually active*	8^a^^,b^				11^b^			.08
.50						16.98%	(7.70%, 33.38%)	
.70						25.30%	(15.40%, 38.65%)	
.90						35.93%	(20.53%, 54.90%)	
*Have consumed alcohol*^c^					14			.88
.25						36.17%	(25.66%, 48.19%)	
.50						35.44%	(24.89%, 47.63%)	
.75						34.72%	(19.67%, 53.61%)	
*Proportion of STD/STI infection*	6^a^				11	.03	(− .00, .06)	.07
.10						26.37%	(16.28%, 39.74%)	
.25						42.24%	(29.16%, 56.50%)	
.50						60.60%	(30.96%, 84.07%)	

“k” = number of studies included, “eff” = effect size (mean prevalence), “95% CI” = 95% confidence interval

Analyses examining quantitative moderator variables, such as the mean age of sexual debut, mean of sexual partners, and the proportion of users of specific substances in the samples (e.g., marijuana, ecstasy, cocaine, ketamine, gamma-hydroxybutyrate, poppers, heroine, lysergic acid diethylamide, methamphetamine, and injecting drugs), were not carried out due to an insufficient number of studies. Likewise, an insufficient number of studies assessing the proportion of sexually active participants in timeframes equal to or less than twelve months was found in order to consider it as moderator

Subgroup analysis could not be performed for the substances marijuana, cocaine, heroin, methamphetamine, ecstasy, poppers and gamma hydroxybutyrate, due to an insufficient number of studies

^a^Insufficient “k” to make comparisons

^b^All included studies evaluated the lifetime prevalence of sexually active individuals in the sample

^c^All included studies evaluated the lifetime prevalence of alcohol consumption of individuals in the sample

#### Alcohol Prevalence Moderators

Prior to describing the results by substance, we would like to emphasize that categorical and quantitative moderators could only be analyzed in the case of alcohol. Subgroup analysis could not be performed for other substances, including marijuana, cocaine, heroin, methamphetamine, ecstasy, poppers, and GHB, due to an insufficient number of studies.

The mean prevalence of alcohol consumption before or during sex among university students was of 31.64% (95% CI: 23.49%, 41.10%). Similar prevalence estimates were obtained from studies considering community samples (44.19%; 95% CI: 26.60%, 63.37%) and migrant workers (35.12%; 95% CI: 15.11%, 62.20%) (*p* = .51) (Table 4). Estimates for clinical and MSM samples could not be performed due to an insufficient number of studies. As no statistically significant differences were observed by sample type, subsequent analyses were performed on the total number of analytical samples.

As indicated in Table 4, the geographical origin and the proportion of ethnic whites in samples significantly moderated the prevalence of using alcohol before or during sex (*p* < .01 for both). The prevalence of having sex under the influence of alcohol in Africa (10.65%; 95% CI: 4.98%, 21.32%) was significantly lower than in Asia (43.07%; 95% CI: 27.93%, 59.62%), Europe (55.46%; 95% CI: 32.74%, 76.10%), and the United States and Canada (36.77%; 95% CI: 28.92%, 45.40%). Moreover, the prevalence of alcohol use before or during sex was higher among studies that sampled a greater proportion of ethnic whites (e.g., 23.44%, 95% CI: 14.26%, 36.05%, when the proportion was .15, versus 43.41%, 95% CI: 32.99%, 54.46%, when it was .85, averaging all studies reviewed) (Table 5).

Results also indicated that men (26.73%; 95% CI: 8.63%, 58.50%) and women (20.77%; 95% CI: 6.34%, 50.38%) consumed alcohol equally before or during sex (*p* = .73). Furthermore, it should be noted that the analysis of the timeframe of the measure was quasi-significant (*p* = .07), showing that studies assessing the prevalence of consuming alcohol before or during sex in timeframes from one to 6 months reported a lower prevalence (24.45%; 95% CI: 14.23%, 38.69%) than studies without timeframes or indicated timeframes in excess of six months (40.51%; 95% CI: 30.34%, 51.58%) (Table 4). The prevalence of alcohol use before or during sex was seen to increase as the proportion of sexually active participants in samples increased (e.g., 16.98%, 95% CI: 7.70%, 33.38%, when the proportion was .50, versus 35.93%, 95% CI: 20.53%, 54.90%, when it was .90), although statistical significance was not reached (*p* = .08). There was a similar relation (*p* = .07) between the prevalence of alcohol use before or during sex and the proportion of STD/STI infections in samples (26.37% when the proportion was .10, 42.24% when it was .25, and 60.60% when it was .50) (Table 5). Prevalence was not moderated by document type, risk of bias, sampling technique, administration procedure, data collection year, proportion of heterosexuals, or sample mean age.

## Discussion

This study provides a systematic review and meta-estimate of the prevalence of the use of intoxicating substances before or during sex among young adults. To the best of our knowledge, this is the first study that specifically examines the prevalence of this behavior in this age grouping. Previous literature has mainly focused on analyzing SDU in MSM communities (Edmundson et al., 2018; Guerra et al., 2020; Íncera-Fernández et al., 2021; Lafortune et al., 2021; Maxwell et al., 2019), which makes it difficult to assess comparability.

Our results have revealed a high global prevalence of the use of intoxicating substances before or during sex, that is, 28–47% of young adults. This high prevalence estimate concurs with those reported in the abovementioned review studies that specifically focused on examining SDU among MSM (Edmundson et al., 2018; Maxwell et al., 2019). Nonetheless, comparisons should be made with caution, considering the differences in the meta-analytical samples. The results obtained in this research also indicate that the prevalence of using an intoxicating substance before or during sex varies significantly according to the specific substance used. The consumption of alcohol, marijuana, and ecstasy had significantly higher prevalences than those referring to the use of cocaine, heroin, methamphetamine, and GHB, perhaps due to differences in their acceptance, accessibility, cost, addictive potential, and short- and medium-term health and social consequences (Bourne et al., 2014; Graupensperger et al., 2021; Jackson et al., 2021; Rosińska et al., 2018). It is also likely that higher prevalence rates and the decision to use one or another substance depends on the particular effects it causes during sex. Alcohol may be used to become disinhibited before sex (Herbenick et al., 2021), even though it may make it more difficult to reach orgasm and reduce quality of sex (George, 2019; Palamar et al., 2018a). Ecstasy, on the other hand, may affect sexual experience the most, for example, by prolonging erection duration (Coyer et al., 2022). Future research should go more deeply into the reasons that lead to the choice of one or another drug, considering the sexual act as a complex process (e.g., from flirtation right through to after-sex behaviors), distinguishing the type and quantity of substances consumed, and the order in which they are consumed, if more than one is involved. The decision to consume one drug or another before or during sex may also be associated with individual characteristics (e.g., conception of sexuality and attitudes towards substance use for pleasure-seeking) and demographic characteristics (e.g., age and work-related aspects, including income level) and, for example, older cohorts may use higher-cost and harder-to-access substances (e.g., methamphetamine and cocaine). Regarding the geographical origin of samples, it was found that the estimated prevalence of alcohol use before or during sex varied significantly and was lowest on the African continent. Geographical comparisons pose a particular challenge due to significant continent-level, country-level, region-level, and population subgroup differences. Though this study’s comparisons are made for descriptive purposes only, several multi-level societal and individual factors may explain the observed differences in alcohol consumption patterns, including economic development, cultural aspects, such as cultural norms and religious beliefs, and societal and political aspects, such as rules concerning drug access and use, and the effectiveness of alcohol control policies (Addo et al., 2018). Future studies may further explore such factors in order to gain more insight into the driving forces behind such variations.

The meta-regression analyses of this study additionally indicate that the prevalence of using intoxicating substances before or during sex differs significantly as a function of the ethnic composition of samples. When considering alcohol, the prevalence was significantly higher in studies with a higher proportion of ethnic whites in their samples. This is in agreement with a previous meta-analysis suggesting that risky sex and drug-use behaviors were reported most frequently by studies that sampled more ethnic whites (Cunningham et al., 2017). However, to our knowledge, reasons for such differences have not been examined yet in the literature. Previous empirical studies have suggested that individual developmental trajectories and socio-cultural and economic factors may help explain ethnic trends and differences in drug use among adolescents and young adults (Chen & Jacobson, 2012; Evans et al., 2017; Vaughn et al., 2018), with white youths having higher rates of illicit drug and alcohol abuse (Johnston et al., 2019; Jones et al., 2020). Future research might explore such aspects in order to improve our understanding of protective and risk exposure factors that may elucidate potential ethnic differences in the use of intoxicating substances before or during sex. However, when considering the global prevalence, a significant inverse relationship is seen. This opposing result is not directly interpretable, since there may be compensatory effects deriving from raw measures that do not distinguish between substances (Cunningham et al., 2017). Other demographic and sexual variables, such as gender, age or sexual orientation, did not show statistically significant effects on the prevalence of intoxicating substance use before or during sex. Regarding gender, for example, the findings of our meta-analysis concur with recent empirical studies suggesting that women present rates of alcohol or other substance use similar to that of men (Ford et al., 2021b; McKetta et al., 2022), although these studies did not focus on consumption before or during sexual activity. Developmental and sociocultural factors may explain the non-difference concerning these variables. For example, young adulthood is a developmental period characterized by the reinforcement of one’s own sexual identity and orientation, in which drugs and sex may be taken as a means of experiencing a variety of sexual practices in common pleasure-seeking (Arnett, 2005, 2007, 2014), regardless of the abovementioned individual participant characteristics (Bourne et al., 2015a; Piyaraj et al., 2018; Schmidt et al., 2016). This, together with previous arguments on the acceptance and accessibility of intoxicating substances in this age group, could offer a potential explanation of the results obtained in this study. Likewise, it should also be taken into account that a large part of our meta-analytical samples are studies with university or community samples, which possess certain common characteristics in their samples (e.g., age range, sexual-developmental stage, educational stage). As several researchers have observed, drug consumption and sex are common occurrences in college contexts and populations (Ford et al., 2021b; McKetta et al., 2022).

In addition to the above, the low quality of both our meta-analytic sample and its measures are an important aspect to highlight in our research. The majority of the studies reviewed had a high risk of bias, employing a cross-sectional design and non-probabilistic sampling techniques, with poor quality measures. The analysis of the measures used in the primary studies is a differential contribution of this review, yielding important gaps that need to be addressed. Indeed, sex under the influence of intoxicating substances appears to have hitherto been measured using “summary measures”, for example, mono-items with dichotomous response options, that not allow researchers to fully characterize the phenomenon (Wells et al., 2015). What may be more important than assessing whether participants have practiced sex under the influence of intoxicating substance or not is the frequency in which they have engaged in such activity. This is because the higher the frequency, the greater the probability of exposure to risks and consequences. Another result to be highlighted is that operational elements such as the specific sexual act (how), relationship type (with who), intentionality (purpose), willingness (whether solicited or not), and drug used were not made explicit in the majority of the studies reviewed. These elements were, indeed, often left up to the interpretation of respondents. The indefiniteness of key operational elements may, therefore, be behind the high dispersion in prevalence estimates. However, regarding drug type, our meta-analytical results do demonstrate that prevalence differences between individual drugs exist. Empirical research has also found that sexual practices under the influence of alcohol or other drugs differ somewhat, with the most frequently reported being exploratory acts (e.g., caressing or touching) (71%) and vaginal penetration (64%), while anal sex (12%) and sex with erotic toys (8%) were the least frequently reported (Castaño et al., 2012). Relevant circumstances such as unprotected sex or relationship type were indicated in only a few studies (Wells et al., 2015), while the willingness of participants was not made explicit in any measure. Nonetheless, it has been found that alcohol consumption before sex increases the probability of unprotected sex and higher-risk sexual encounters (i.e., with non-primary partners), increasing the likelihood of exposure to STIs and other risks (Rizwan et al., 2014). It should be noted that, although the result was not statistically significant, the proportion of STD/STI infections in the samples increased as the proportion of the use of alcohol before or during sex increased. Another key aspect is the specification of intentionality and purpose involved in the use of intoxicating substances before or during sex. Both are key indicators when differentiating between planned substance use with the purpose of enhancing sexual experience (e.g., SDU, chemsex) from substance use preceding casual sexual activity. All such measure quality related aspects reasonably warrant the wide credibility/prediction intervals obtained in our study. The lack of similar operationalizations with a minimum of precision makes it difficult to perform plausible comparisons among studies. Thus, clarifying these elements in operational definitions is a priority for future research that will allow a distinction to be made between planned and unplanned drug use before or during sex.

### Educational Implications

Based on the results of this study and the prevalence of the use of intoxicating substances before or during sex, developing educational prevention programs referring to substance abuse and sexual risks remains imperative. Research recommends that such programs should revolve around four fundamental principles. Firstly, preventative efforts should be approached from an integrative perspective that contemplates biopsychological, social-communitarian and sexual aspects through multidisciplinary support (Donnadieu-Rigole et al., 2020). In particular, based on the results of the present study, educational interventions should take into account social-communitarian characteristic of participants, such as geographical and ethnic origins (e.g., focusing on more disadvantaged or higher risk populations, or on areas with higher SDU prevalence rates). Secondly, educational measures should take into account the implications, that is, the effects and consequences, of different substances and types of sexual practices (Lafortune et al., 2021; Saengdidtha et al., 2016). This is emphasized in studies that associate, for instance, substance use before or during sexual activity with negative health outcomes such as overdoses (Hammoud et al., 2017; Hegazi et al., 2017) or risky sexual practices such as unprotected sex (D’Anna et al., 2021; Ristuccia et al., 2018). Parents and educators should also inform young people on the implications and sexual health risks involved in having sexual experiences with someone you know personally, as opposed to someone met more casually. Thirdly, information on ethical issues relating to willingness and consent should be provided (Muehlenhard et al., 2016). Fourthly, young people might be encouraged to make greater use of sexual health resources if interventions were to focus more on the enjoyment of safe sex and sexual pleasure-seeking, in addition to discussing sexual risk behaviors (Ford et al., 2021a). Perhaps, a good starting point for educational interventions aimed at addressing SDU would be an analysis of needs and motivations that lead young adults to use intoxicating substances before or during sex. If the pursuit of pleasure emerges as the main motivation, it would, therefore, be a fundamental element to take into account in order to improve and adapt sexual health interventions. Interventions informing youths about decisions concerning where (e.g., spaces), how (e.g., substance characteristics in terms of effects and consequences; information on contraceptive methods; management of sexual acts and potential implications) and with whom they have sex may help them both to be more fully aware of their sexual practices and to avoid negative sexual experiences and adverse health outcomes. Ideally, such educational measures should be introduced at fairly early ages. Although it was non-significant, results of this meta-analysis showed that rates of alcohol use before or during sex increased as the proportion of sexually active participants in samples increased. Thus, it would be logical to start intervening at ages when young people begin to experiment with drugs and have their first sexual relations, with continuity spreading across into other educational courses and contexts, such as compulsory secondary education and university, and beyond school curricula, through ad hoc preventative educational programs, accessible community services, and information campaigns.

### Limitations

This study has certain evident limitations, the main one being the low quality meta-analytical sample. Indeed, the predominant use of observational designs, non-probabilistic sampling techniques and non-validated measures was seen to introduce high levels of risk of bias in the primary studies. Secondly, the meta-analytical results showed a significant between-study variability, only partially explained by certain moderator variables. Undeniably, a considerable part of such variability remains unexplained and thus interpretable by means of the set of moderators and covariates considered. The high heterogeneity may also be attributed to a variety of other aspects such as research objectives, sample sizes, sample population characteristics and the specific measures used. Thirdly, several reviewed articles provided only a crude prevalence regarding the use of intoxicating substances before or during sex, without specifying the particular substance used, forcing us to analyze this as a generalized proxy. However, such results should be taken with caution because they may be biased and not necessarily reflect the effective prevalence rate. Fourthly, it was not possible to estimate the prevalence of the use of certain substances such as crack, speed, sedatives, LSD and ketamine before or during sex due to insufficient data. Furthermore, in the case of the global prevalence and of the use of alcohol, it was not possible to examine the effect of certain categorical and quantitative moderators due to an insufficient number of studies at some level. Fifthly, socioeconomic status could not be analyzed as a moderating variable due to deficiencies and divergences in how empirical studies have recorded and reported it (e.g., self-reported income level, social class, education level, occupation). Yet another limitation of this meta-analysis is that, despite including studies from a variety of countries, certain regions were particularly underrepresented. Data from developing countries and non-occidental countries were scarce. Lastly, because only 5 studies reported prevalence data of at least two or more substances, due to software limitations, it was not possible to compute a multi-level/multivariate meta-analysis assuming a generalized linear model. Developing this possibility would allow us to simultaneously analyze data from studies with multiple non-normally distributed outcomes, taking into account the dependence among effect sizes from the same study. Given all the above limitations, the generalizability of the meta-analytical results should be considered with caution.

### Conclusions

In conclusion, our meta-analysis results suggest a high mean prevalence of intoxicating substance use before or during sex among young adults. The prevalence was highly heterogeneous, and moderator analysis revealed the geographical origin and ethnic composition of the sample as significant moderators. The results also highlighted the great difficulties in accurately determining the prevalence of this behavior, underlining the importance of introducing more rigorous, consistent, and reliable measures to reduce potential bias. We, therefore, believe that the results make a valuable contribution to improving research in this area, particularly in the design of more suitable operational definitions, and more reliable and valid measurement procedures. Higher quality measures will consequently allow more accurate prevalence estimates, and a more complete characterization of this behavior among young adults.

### Electronic supplementary material

Below is the link to the electronic supplementary material.

Supplementary file1 (DOCX 20 KB)

Supplementary file2 (DOCX 52 KB)

Supplementary file3 (DOCX 37 KB)

Supplementary file4 (DOCX 32 KB)

## Data Availability

The datasets generated and analyzed in this study are provided as supplementary files. All materials will be publicly available.

## Appendix 1

PRISMA 2020 for Abstracts Checklist

**Table:**

Section and topic	Item #	Checklist item	Reported (Yes/No)
*Title*			
Title	1	Identify the report as a systematic review	Yes
*Background*			
Objectives	2	Provide an explicit statement of the main objective(s) or question(s) the review addresses	Yes
*Methods*			
Eligibility criteria	3	Specify the inclusion and exclusion criteria for the review	Yes
Information sources	4	Specify the information sources (e.g. databases, registers) used to identify studies and the date when each was last searched	Yes
Risk of bias	5	Specify the methods used to assess risk of bias in the included studies	Yes
Synthesis of results	6	Specify the methods used to present and synthesise results	Yes
*Results*			
Included studies	7	Give the total number of included studies and participants and summarise relevant characteristics of studies	Yes
Synthesis of results	8	Present results for main outcomes, preferably indicating the number of included studies and participants for each. If meta-analysis was done, report the summary estimate and confidence/credible interval. If comparing groups, indicate the direction of the effect (i.e. which group is favoured)	Yes
*Discussion*			
Limitations of evidence	9	Provide a brief summary of the limitations of the evidence included in the review (e.g. study risk of bias, inconsistency and imprecision)	Yes
Interpretation	10	Provide a general interpretation of the results and important implications	Yes
*Other*			
Funding	11	Specify the primary source of funding for the review	See funding details
Registration	12	Provide the register name and registration number	See full-text

From: Page, M. J., McKenzie, J. E., Bossuyt, P. M., Boutron, I., Hoffmann, T. C., Mulrow, C. D., Shamseer, L. … Moher, D. (2021). The PRISMA 2020 statement: An updated guideline for reporting systematic reviews*. BMJ*, *372*(71). 10.1136/bmj.n71

For more information, visit: http://www.prisma-statement.org/

## Appendix 2

PRISMA 2020 Checklist

**Table:**

Section and topic	Item #	Checklist item	Location where item is reported
*Title*			
Title	1	Identify the report as a systematic review	Pg. 1
*Abstract*			
Abstract	2	See the PRISMA 2020 for Abstracts checklist	Completed
*Introduction*			
Rationale	3	Describe the rationale for the review in the context of existing knowledge	Pg. 2–5
Objectives	4	Provide an explicit statement of the objective(s) or question(s) the review addresses	Pg. 5–6
*Methods*			
Eligibility criteria	5	Specify the inclusion and exclusion criteria for the review and how studies were grouped for the syntheses	Pg. 7
Information sources	6	Specify all databases, registers, websites, organisations, reference lists and other sources searched or consulted to identify studies. Specify the date when each source was last searched or consulted	Pg. 6
Search strategy	7	Present the full search strategies for all databases, registers and websites, including any filters and limits used	Sup. F. 2
Selection process	8	Specify the methods used to decide whether a study met the inclusion criteria of the review, including how many reviewers screened each record and each report retrieved, whether they worked independently, and if applicable, details of automation tools used in the process	Pg. 7
Data collection process	9	Specify the methods used to collect data from reports, including how many reviewers collected data from each report, whether they worked independently, any processes for obtaining or confirming data from study investigators, and if applicable, details of automation tools used in the process	Pg. 7–8
Data items	10a	List and define all outcomes for which data were sought. Specify whether all results that were compatible with each outcome domain in each study were sought (e.g. for all measures, time points, analyses), and if not, the methods used to decide which results to collect	Pg. 7–9
	10b	List and define all other variables for which data were sought (e.g. participant and intervention characteristics, funding sources). Describe any assumptions made about any missing or unclear information	Pg. 7–9
Study risk of bias assessment	11	Specify the methods used to assess risk of bias in the included studies, including details of the tool(s) used, how many reviewers assessed each study and whether they worked independently, and if applicable, details of automation tools used in the process	Pg. 8–9
Effect measures	12	Specify for each outcome the effect measure(s) (e.g. risk ratio, mean difference) used in the synthesis or presentation of results	Pg. 9
Synthesis methods	13a	Describe the processes used to decide which studies were eligible for each synthesis (e.g. tabulating the study intervention characteristics and comparing against the planned groups for each synthesis (item #5))	Pg. 7
	13b	Describe any methods required to prepare the data for presentation or synthesis, such as handling of missing summary statistics, or data conversions	Pg. 7–9
	13c	Describe any methods used to tabulate or visually display results of individual studies and syntheses	Pg. 8–9
	13d	Describe any methods used to synthesize results and provide a rationale for the choice(s). If meta-analysis was performed, describe the model(s), method(s) to identify the presence and extent of statistical heterogeneity, and software package(s) used	Pg. 9
	13e	Describe any methods used to explore possible causes of heterogeneity among study results (e.g. subgroup analysis, meta-regression)	Pg. 9
	13f	Describe any sensitivity analyses conducted to assess robustness of the synthesized results	Not applicable
Reporting bias assessment	14	Describe any methods used to assess risk of bias due to missing results in a synthesis (arising from reporting biases)	Not applicable
Certainty assessment	15	Describe any methods used to assess certainty (or confidence) in the body of evidence for an outcome	Pg. 6–9
*Results*			
Study selection	16a	Describe the results of the search and selection process, from the number of records identified in the search to the number of studies included in the review, ideally using a flow diagram	Pg. 10
	16b	Cite studies that might appear to meet the inclusion criteria, but which were excluded, and explain why they were excluded	Sup. F 3 and 4
Study characteristics	17	Cite each included study and present its characteristics	Sup. F 5
Risk of bias in studies	18	Present assessments of risk of bias for each included study	Sup. F 7
Results of individual studies	19	For all outcomes, present, for each study: (a) summary statistics for each group (where appropriate) and (b) an effect estimate and its precision (e.g. confidence/credible interval), ideally using structured tables or plots	Table 3 and Forest plots
Results of syntheses	20a	For each synthesis, briefly summarise the characteristics and risk of bias among contributing studies	Table 1
	20b	Present results of all statistical syntheses conducted. If meta-analysis was done, present for each the summary estimate and its precision (e.g. confidence/credible interval) and measures of statistical heterogeneity. If comparing groups, describe the direction of the effect	Table 3
	20c	Present results of all investigations of possible causes of heterogeneity among study results	Tables 4 and 5
	20d	Present results of all sensitivity analyses conducted to assess the robustness of the synthesized results	Not applicable
Reporting biases	21	Present assessments of risk of bias due to missing results (arising from reporting biases) for each synthesis assessed	Not applicable
Certainty of evidence	22	Present assessments of certainty (or confidence) in the body of evidence for each outcome assessed	Pg. 11–13
*Discussion*			
Discussion	23a	Provide a general interpretation of the results in the context of other evidence	Pg. 15–16
	23b	Discuss any limitations of the evidence included in the review	Pg. 18
	23c	Discuss any limitations of the review processes used	Pg. 21–22
	23d	Discuss implications of the results for practice, policy, and future research	Pg. 15–20
*Other information*			
Registration and protocol	24a	Provide registration information for the review, including register name and registration number, or state that the review was not registered	Pg. 6
	24b	Indicate where the review protocol can be accessed, or state that a protocol was not prepared	Pg. 6
	24c	Describe and explain any amendments to information provided at registration or in the protocol	Not applicable
Support	25	Describe sources of financial or non-financial support for the review, and the role of the funders or sponsors in the review	See funding information
Competing interests	26	Declare any competing interests of review authors	See competing interest’s information
Availability of data, code and other materials	27	Report which of the following are publicly available and where they can be found: template data collection forms; data extracted from included studies; data used for all analyses; analytic code; any other materials used in the review	See supplementary files

From: From: Page, M. J., McKenzie, J. E., Bossuyt, P. M., Boutron, I., Hoffmann, T. C., Mulrow, C. D., Shamseer, L. …Moher, D. (2021). The PRISMA 2020 statement: An updated guideline for reporting systematic reviews. *BMJ*, *372*(71). 10.1136/bmj.n71

For more information, visit: http://www.prisma-statement.org/

## Appendix 3

*References of Included Studies*

Antón Ruiz, F. A., & Espada, J. P. (2009). Consumo de sustancias y conductas sexuales de riesgo para la transmisión del VIH en una muestra de estudiantes universitarios. *Anales de Psicologia*, *25*(2), 344–350.

Apostolopoulos, Y., Sönmez, S., & Yu, C. H. (2002). HIV-risk behaviours of American spring break vacationers: A case of situational disinhibition? *International Journal of STD and AIDS*, *13*(11), 733–743. 10.1258/095646202320753673

Aung, H. P., & Panza, A. (2016). Factors influencing sexual behaviors among young Myanmar migrant workers in Samut Sakhon, Thailand. *Journal of Health Research*, *30*(Suppl.1), S45–S51. 10.14456/jhr.2016.66

Baćak, V., & Štulhofer, A. (2012). Condom use errors and problems in a national sample of young Croatian adults. *Archives of Sexual Behavior*, *41*(4), 995–1003. 10.1007/s10508-011-9838-x

Bianchi, G., & Popper, M. (2000). Interaction of substance use and risks to sexual health in the Slovak army: General, sociocultural and individual behaviour patterns. *AIDS Care—Psychological and Socio-Medical Aspects of AIDS/HIV*, *12*(6), 757–765. 10.1080/09540120020014309

Boone, T. L., & Lefkowitz, E. S. (2004). Safer sex and the health belief model: Considering the contributions of peer norms and socialization factors. *Journal of Psychology and Human Sexuality*, *16*(1), 51–68. 10.1300/J056v16n01_04

Braithwaite, S. R., Givens, A., Brown, J., & Fincham, F. (2015). Is pornography consumption associated with condom use and intoxication during hookups? *Culture, Health and Sexuality*, *17*(10), 1155–1173. 10.1080/13691058.2015.1042920

Brown, E. J. (2000). AIDS-related risk behavior of young college students. *The ABNF Journal, 11*(2), 37–43.

Brown, J. L., & Vanable, P. A. (2007). Alcohol use, partner type, and risky sexual behavior among college students: Findings from an event-level study. *Addictive Behaviors*, *32*(12), 2940–2952. 10.1016/j.addbeh.2007.06.011

Chirinda, W., & Peltzer, K. (2014). Correlates of inconsistent condom use among youth aged 18-24 years in South Africa. *Journal of Child and Adolescent Mental Health*, *26*(1), 75–82. 10.2989/17280583.2013.877912

Choi, K. H., Operario, D., Gregorich, S. E., McFarland, W., MacKellar, D., & Valleroy, L. (2005). Substance use, substance choice, and unprotected anal intercourse among young Asian American and Pacific Islander men who have sex with men. *AIDS Education and Prevention*, *17*(5), 418–429. 10.1521/aeap.2005.17.5.418

Christian, M. D. (2001). *Consequences of sexual self-concept discrepancies in African American women* [Doctoral dissertation, The University of Michigan]. ProQuest Dissertations & Theses Global. https://www.proquest.com/openview/50fad06f538d257f8116717107169d57/1?pq-origsite=gscholar&cbl=18750&diss=y

Cohan, D. L., Kim, A., Ruiz, J., Morrow, S., Reardon, J., Lynch, M., Klausner, J. D., Molitor, F., Allen, B., Ajufo, B. G., Ferrero, D., Sanford, G. B., Page-Shafer, K., Delgado, V., & McFarland, W. (2005). Health indicators among low-income women who report a history of sex work: The population based Northern California Young Women’s Survey. *Sexually Transmitted Infections*, *81*(5), 428–433. 10.1136/sti.2004.013482

D’Anna, L. H., Chang, K., Wood, J., & Washington, T. A. (2021). Marijuana use and sexual risk behavior among young black men who have sex with men in California. *Journal of Racial and Ethnic Health Disparities, 87*(6), 1522–1532. 10.1007/s40615-020-00915-3

Des Rosiers, S. E., Schwartz, S. J., Zamboanga, B. L., Ham, L. S., & Huang, S. (2013). A cultural and social cognitive model of differences in acculturation orientations, alcohol expectancies, and alcohol-related risk behaviors among Hispanic college students. *Journal of Clinical Psychology*, *69*(4), 319–340. 10.1002/jclp.21859

Dong, Y., Zhang, H., Wang, Y., Tao, H., Xu, S., Xia, J., Huang, W., He, H., Zaller, N., & Operario, D. (2015). Multiple abortions and sexually transmitted infections among young migrant women working in entertainment venues in China. *Women and Health*, *55*(5), 580–594. 10.1080/03630242.2015.1022811

Fantasia, H. C., Fontenot, H. B., Sutherland, M. A., & Lee-St. John, T. J. (2015). Forced sex and sexual consent among college women. *Journal of Forensic Nursing*, *11*(4), 223–231. 10.1097/JFN.0000000000000086

Feinstein, B. A., Moody, R. L., John, S. A., Parsons, J. T., & Mustanski, B. (2018). A three-city comparison of drug use and drug use before sex among young men who have sex with men in the United States. *Journal of Gay and Lesbian Social Services*, *30*(1), 82–101. 10.1080/10538720.2018.1408519

Guimarães, M. D. C., Elkington, K. S., Gomes, A. L. F. M., Veloso, C., & McKinnon, K. (2014). HIV sexual risk behavior among emerging adults in psychiatric treatment in Brazil. *Journal of HIV/AIDS and Social Services*, *13*(4), 451–472. 10.1080/15381501.2013.809042

Hamilton, K. M., Falletta, L., Fischbein, R., & Kenne, D. R. (2019). Non-medical use of prescription drugs during sexual activity as a predictor of condom use among a sample of college students. *Journal of American College Health*, *67*(5), 459–468. 10.1080/07448481.2018.1486843

Hoque, M. E., & Ghuman, S. (2011). Sexual behaviour and knowledge regarding sexually transmitted infections among undergraduate students in Durban, South Africa. *Gender and Behaviour*, *9*(1), 3710–3728. 10.4314/gab.v9i1.67469

Jackson, D. D. (2014). *The development, implementation, and testing of an interactive sexual health web-based application intervention to reduce sexual risk behaviors among college students* [Doctoral dissertation, University of Maryland]. Scholar Commons. https://scholarcommons.sc.edu/etd/2845/

Jones, R. (2001). *The relations of dyadic trust, sensation-seeking, and sexual imposition with sexual risk behaviors in young, urban women with primary and non-primary male partners* [Doctoral dissertation, New York University]. ProQuest Dissertations & Theses Global. https://www.proquest.com/dissertations-theses/relations-dyadic-trust-sensation-seeking-sexual/docview/619943615/se-2?accountid=14777%0Ahttps://uv.primo.exlibrisgroup.com/openurl/34CVA_UV/34CVA_UV:VU1??url_ver=Z39.88-2004&rft_val_fmt=info:ofi/fmt:kev:mtx:d

Jones, R., & Hoover, D. R. (2018). Reduction in a high-risk sex script among young urban women in the Love, Sex, & Choices web video HIV prevention intervention study. *Research in Nursing and Health*, *41*(6), 535–543. 10.1002/nur.21912

Kim, J. L., Schooler, D. E., Lazaro, S. K., & Weiss, J. (2019). Watching reality TV programs with concurrent sexual and alcohol themes is associated with risky drinking and sexual experiences. *Emerging Adulthood*, *7*(1), 59–65. 10.1177/2167696818754920

Kim, S., De La Rosa, M., Trepka, M. J., & Kelley, M. (2007). Condom use among unmarried students in a Hispanic-serving university. *AIDS Education and Prevention*, *19*(5), 448–461. 10.1521/aeap.2007.19.5.448

Kogan, S. M., Cho, J., Barnum, S. C., & Brown, G. L. (2015). Correlates of concurrent sexual partnerships among young, rural African American men. *Public Health Reports*, *130*(4), 392–399. 10.1177/003335491513000418

Loza, O., Mangadu, T., Ferreira-Pinto, J. B., & Guevara, P. (2021). Differences in substance use and sexual risk by sexual orientation and gender identity among university and community young adults in a U.S.-Mexico border city. *Health Promotion Practice*, *22*(4), 559–573. 10.1177/1524839920933257

Makgale, O. L., & Plattner, I. E. (2017). Sexting and risky sexual behaviours among undergraduate students in Botswana: An exploratory study. *Cyberpsychology: Journal of Psychosocial Research on Cyberspace*, *11*(2), Article 1. 10.5817/CP2017-2-1

Mayo-Wilson, L. J., Mathai, M., Yi, G., Mak’anyengo, M. O., Davoust, M., Massaquoi, M. L., Baral, S., Ssewamala, F. M., Glass, N. E., & NAHEDO Study Group. (2020). Lessons learned from using respondent-driven sampling (RDS) to assess sexual risk behaviors among Kenyan young adults living in urban slum settlements: A process evaluation. *PLoS ONE*, *15*(4), e0231248. 10.1371/journal.pone.0231248

Mcharo, R. D., Olomi, W., Mayaud, P., & Msuya, S. E. (2021). Risky sexual behaviours among young adults attending higher learning institutions in Mbeya, Tanzania: Implications for STIs and HIV preventive programs. *AAS Open Research*, *3*. 10.12688/aasopenres.13123.1

Merianos, A. L., King, K. A., & Vidourek, R. A. (2013). Body image satisfaction and involvement in risky sexual behaviors among university students. *Sexuality and Culture*, *17*(4), 617–630. 10.1007/s12119-013-9165-6

Metzger, I. W. (2015). *Profiles of African American college students’ risky behaviors: General and culturally-specific stress and social support as factors of risk and resilience?* [Doctoral dissertation, University of South Carolina - Columbia]. ProQuest Dissertations & Theses Global. http://pitt.idm.oclc.org/login?url=https://www.proquest.com/dissertations-theses/profiles-african-american-college-students-risky/docview/1702157714/se-2?accountid=14709%0Ahttps://pitt.primo.exlibrisgroup.com/openurl/01PITT_INST/01PITT_INST:01PITT_INST??u

Meuwly, M., Auderset, D., Stadelmann, S., Suris, J. C., & Barrense-Dias, Y. (2021). Anal intercourse among heterosexual young adults: A population-based survey in Switzerland. *Journal of Sex Research*, *58*(8), 1061–1068. 10.1080/00224499.2020.1866481

Miller, J. D., Lynam, D., Zimmerman, R. S., Logan, T. K., Leukefeld, C., & Clayton, R. (2004). The utility of the five factor model in understanding risky sexual behavior. *Personality and Individual Differences*, *36*(7), 1611–1626. 10.1016/j.paid.2003.06.009

Neuman, M. E., Simonovich, S. D., & Amer, K. (2019). Exploring the protective effects of Judaism on risky behaviors in college students: A pilot study. *Journal of Pediatric Nursing*, *49*, 79–84. 10.1016/j.pedn.2019.09.003

Otiniano, A. D., Dyer, T. P., Friedman, S. R., & Gee, G. C. (2020). Discrimination and sexual risk among Caribbean Latinx young adults. *Ethnicity and Health*, *25*(5), 639–652. 10.1080/13557858.2018.1444148

Palamar, J. J., Griffin-Tomas, M., Acosta, P., Ompad, D. C., & Cleland, C. M. (2018). A comparison of self-reported sexual effects of alcohol, marijuana, and ecstasy in a sample of young adult nightlife attendees. *Psychology and Sexuality*, *9*(1), 54–68. 10.1080/19419899.2018.1425220

Peterson, L. M. (2013). *Impact of television media depicting sex under the influence of alcohol on cognitions derived from the prototype willingness model* [Doctoral dissertation, The George Washington University]. GW Scholar Space. https://scholarspace.library.gwu.edu/concern/gw_etds/bc386j29t?locale=es

Powell, E. M. (2018). *Exploring the interrelationships among college students’ attachment orientations, sexual motives, and sexual risk-taking* [Doctoral dissertation, University of Houston]. UH Institutional Repository. https://uh-ir.tdl.org/handle/10657/3357

Reid, G. J., Siu, S. C., McCrindle, B. W., Irvine, M. J., & Webb, G. D. (2008). Sexual behavior and reproductive concerns among adolescents and young adults with congenital heart disease. *International Journal of Cardiology*, *125*(3), 332–338. 10.1016/j.ijcard.2007.02.040

Ristuccia, A., LoSchiavo, C., Halkitis, P. N., & Kapadia, F. (2018). Sexualised drug use among sexual minority young adults in the United States: The P18 Cohort Study. *International Journal of Drug Policy*, *55*, 207–214. 10.1016/j.drugpo.2018.03.014

Rizwan, S., Kant, S., Goswami, K., Rai, S., & Misra, P. (2014). Influence of alcohol on condom use pattern during non-spousal sexual encounter in male migrant workers in north India. *Journal of Postgraduate Medicine*, *60*(3), 276–281. 10.4103/0022-3859.138752

Roberts, S. T., & Kennedy, B. L. (2006). Why are young college women not using condoms? Their perceived risk, drug use, and developmental vulnerability may provide important clues to sexual risk. *Archives of Psychiatric Nursing*, *20*(1), 32–40. 10.1016/j.apnu.2005.08.008

Saengdidtha, B., Rangsin, R., Kaoaiem, H., & Sathityudhakarn, O. (2016). Risk factors for HIV infection among Thai young men aged 21-23 years. *Epidemiology: Open Access*, *6*(3), 248. 10.4172/2161-1165.1000248

Santos, M. J., Ferreira, E., Duarte, J., & Ferreira, M. (2018). Risk factors that influence sexual and reproductive health in Portuguese university students. *International Nursing Review*, *65*(2), 225–233. 10.1111/inr.12387

Sawyer, A. N., Smith, E. R., & Benotsch, E. G. (2018). Dating application use and sexual risk behavior among young adults. *Sexuality Research and Social Policy*, *15*(2), 183–191. 10.1007/s13178-017-0297-6

Schwartz, S. J., Waterman, A. S., Vazsonyi, A. T., Zamboanga, B. L., Whitbourne, S. K., Weisskirch, R. S., Vernon, M., Caraway, S. J., Kim, S. Y., Forthun, L. F., Donnellan, M. B., & Ham, L. S. (2011). The association of well-being with health risk behaviors in college-attending young adults. *Applied Developmental Science*, *15*(1), 20–36. 10.1080/10888691.2011.538617

Scott-Sheldon, L. A. J., Carey, K. B., & Carey, M. P. (2008). Health behavior and college students: Does Greek affiliation matter? *Journal of Behavioral Medicine*, *31*(1), 61–70. 10.1007/s10865-007-9136-1

Snipes, D. J., & Benotsch, E. G. (2013). High-risk cocktails and high-risk sex: Examining the relation between alcohol mixed with energy drink consumption, sexual behavior, and drug use in college students. *Addictive Behaviors*, *38*(1), 1418–1423. 10.1016/j.addbeh.2012.07.011

So, D. W., Wong, F. Y., & DeLeon, J. M. (2005). Sex, HIV risks, and substance use among Asian American college students. *AIDS Education and Prevention*, *17*(5), 457–468. 10.1521/aeap.2005.17.5.457

Tan, R. K. J., O’Hara, C. A., Koh, W. L., Le, D., Tan, A., Tyler, A., Tan, C., Kwok, C., Banerjee, S., & Wong, M. L. (2021). Delineating patterns of sexualized substance use and its association with sexual and mental health outcomes among young gay, bisexual and other men who have sex with men in Singapore: A latent class analysis. *BMC Public Health*, *21*(1), 1026. 10.1186/s12889-021-11056-5

Vail-Smith, K., Maguire, R. L., Brinkley, J., & Burke, S. (2010). Sexual behaviors during the first year of college: An exploratory comparison of first and second semester freshmen. *American Journal of Sexuality Education*, *5*(2), 171–188. 10.1080/15546128.2010.491064

Villegas-Pantoja, M. A., Mendez-Ruiz, M. D., Sánchez-López, L., Herrera-Paredes, J. M., & Álvarez-Aguirre, A. (2022). Harmful use of alcohol as predictor of presex drinking in Mexican young college women. *Journal of Addictions Nursing,* *33*, E52–E59. 10.1097/jan.0000000000000411

Walsh, K., Choo, T. H., Wall, M., Hirsch, J. S., Ford, J., Santelli, J. S., Gilbert, L., Thompson, M. P., Reardon, L., & Mellins, C. A. (2020). Repeat sexual victimization during college: Prevalence and psychosocial correlates. *Psychology of Violence*, *10*(6), 676–686. 10.1037/vio0000339

## Code Availability

R-code is available upon request from the authors.
