# Supplementary material for: Prevalence of Intoxicating Substance Use Before or During Sex Among Young Adults: A Systematic Review and Meta-Analysis
Source: Arch Sex Behav. 2023 Mar 10;52(6):2503–26. doi: 10.1007/s10508-023-02572-z (PMC10501956; doi:10.1007/s10508-023-02572-z)
Supplement: Supplementary file 1 — Supplementary file1 (DOCX 20 KB) [file 10508_2023_2572_MOESM1_ESM.docx]

**Supplementary Table S2a**

*Web of Science search strategy*

| Step Search terms | |
| --- | --- |
| 1 | TS=(youth*) OR TS=(young*) OR TS=(student*) OR TS=("young adult*") OR TS=(juvenil*) OR TS=("emerging adult*") OR TS=("young people") OR TS=("young student*") OR TS=("university student*") OR TS=(universit*) OR TS=(college ) OR TS=(undergraduat*) OR TS=(postgraduat*) OR TS=(graduat*) OR TS=(postsecondary) OR TS=("higher education") OR TS=("tertiary education") OR TS=(bachelor*) OR TS=("master* degree*") OR TS=("doctoral degree*") OR TS=(freshman ) OR TS=("early adult*") OR TS=(adulthood) OR TS=(college-age*) OR TS=("late adolescen*") OR TS=("older adolescen*") OR TS=("junior college") OR TS=("college senior*") OR TS=("18 to 29") OR TS=("18-29") |
| 2 | TS=(chemsex) OR TS=("sex* drug* use") OR TS=("sex* drug* tak*") OR TS=("drug* facilitat* sex*") OR TS=("alcohol facilitat* sex*") OR TS=("drug* enhanc* sex*") OR TS=("alcohol enhanc* sex*") OR TS=("consum* before sex*") OR TS=( "drug* before sex*") OR TS=("drug* use before sex*") OR TS=("alcohol use before sex*") OR TS=("alcohol before sex*") OR TS=("sex after consum*") OR TS=("sex under the influence") OR TS=("sex while us* drug*") OR TS=("sex* while under the influence") OR TS=("sex while drunk*") OR TS=("sex while high") OR TS=("drug* prior to sex*") OR TS=("alcohol prior to sex*") OR TS=("consum* prior to sex*") OR TS=("sex after inject*") OR TS=("sex while intoxicat*") OR TS=("drug* during sex*") OR TS=("alcohol during sex*") OR TS=("alcohol use during sex*") OR TS=("alcohol consum* during sex*") OR TS=("drug* use during sex*") OR TS=("intak* before sex*") OR TS=("intak* during sex*") OR TS=(slamming) OR TS=(slamsex) OR TS=("sex* inject* drug use") OR TS=("inject* during sex*") OR TS=("inject* sex* drug*") OR TS=("sex* after us*") OR TS=("Party-N-Play") OR TS=("Party and Play") OR TS=("intercourse while intoxicat*") OR TS=("intercourse under the influence") OR TS=("intercourse while drunk*") OR TS=("intercourse while high") OR TS=("intercourse while under the influence") OR TS=("intercourse while drink*") OR TS=("sex while drink*") OR TS=("sex* inject* drug*") OR TS=("drug* for sex*") OR TS=("alcohol for sex*") OR TS=("sexual practic* while intoxicat*") OR TS=("sexual practic* under the influence") OR TS=("sexual practic* while under the influence") OR TS= ("drug* us* prior to sex*") OR TS= ("alcohol us* prior to sex*") OR TS= ("sexual* substanc* us*") OR TS= ("substanc* during sex*") OR TS= ("substanc* us* at last sex") OR TS= ("substanc* us* before sex*") OR TS= ("substanc* us* prior to sex*") |
| 3 | 1 AND 2 |
| 4 | FILTER: Publication Year (2000-2021) |
| 5 | FILTER: Language (English or Spanish or Unspecified) |
| 6 | FILTER: Exclude – Document Types (Meeting or Art and Literature or Letters or Reference Material or Patent or Data Papers or News or Books) |

**Supplementary Table S2b**

*PsycINFO search strategy*

| Step Search terms | |
| --- | --- |
| 1 | (if(youth* OR young* OR teen* OR student* OR ("young adult" OR "young adulthood" OR "young adults") OR juvenil* OR ("emerging adult" OR "emerging adulthood" OR "emerging adults") OR "young people" OR ("young student" OR "young students") OR ("university student" OR "university students") OR universit* OR college OR undergraduat* OR postgraduat* OR graduat* OR postsecondary OR "higher education" OR "tertiary education" OR bachelor* OR "master* degree*" OR "doctoral degree" OR freshman OR ("early adult" OR "early adulthood") OR "adulthood" OR ("college age" OR "college aged") OR ("late adolescence" OR "late adolescent" OR "late adolescents") OR ("older adolescent" OR "older adolescents") OR "junior college" OR ("college senior" OR "college seniors") OR "18 to 29") OR ab(youth* OR young* OR teen* OR student* OR ("young adult" OR "young adulthood" OR "young adults") OR juvenil* OR ("emerging adult" OR "emerging adulthood" OR "emerging adults") OR "young people" OR ("young student" OR "young students") OR ("university student" OR "university students") OR universit* OR college OR undergraduat* OR postgraduat* OR graduat* OR postsecondary OR "higher education" OR "tertiary education" OR bachelor* OR "master* degree*" OR "doctoral degree" OR freshman OR ("early adult" OR "early adulthood") OR "adulthood" OR ("college age" OR "college aged") OR ("late adolescence" OR "late adolescent" OR "late adolescents") OR ("older adolescent" OR "older adolescents") OR "junior college" OR ("college senior" OR "college seniors") OR "18 to 29") OR ti(youth* OR young* OR teen* OR student* OR ("young adult" OR "young adulthood" OR "young adults") OR juvenil* OR ("emerging adult" OR "emerging adulthood" OR "emerging adults") OR "young people" OR ("young student" OR "young students") OR ("university student" OR "university students") OR universit* OR college OR undergraduat* OR postgraduat* OR graduat* OR postsecondary OR "higher education" OR "tertiary education" OR bachelor* OR "master* degree*" OR "doctoral degree" OR freshman OR ("early adult" OR "early adulthood") OR "adulthood" OR ("college age" OR "college aged") OR ("late adolescence" OR "late adolescent" OR "late adolescents") OR ("older adolescent" OR "older adolescents") OR "junior college" OR ("college senior" OR "college seniors") OR "18 to 29")) |
| 2 | ((Chemsex OR (alcohol PRE/4 "before sex*") OR "consum* prior to sex*" OR "sex* drug* us*" OR (drug* PRE/2 "enhanc* sex*") OR (alcohol PRE/2 "enhanc* sex*") OR "consum* before sex*" OR "alcohol facilitat* sex*" OR (drug* PRE/4 "before sex*") OR (sex* PRE/4 "under the influence") OR "sex while drunk" OR "sex while high" OR "sex while intoxicat*" OR "sex while us* drug*" OR (drug* PRE/3 "prior to sex*") OR (alcohol PRE/3 "prior to sex*") OR (sex* PRE/3 "after consum*") OR (drug* PRE/4 "during sex*") OR (alcohol PRE/4 "during sex*") OR Slamming OR Slamsex OR "sex* inject* drug use" OR (inject* PRE/4 "during sex*") OR (inject* PRE/4 "sex* drug*") OR "Party-N-Play" OR "Party and Play" OR (sex* PRE/4 "after us*") OR "sex* drug* tak*" OR "intercourse* while intoxicated") OR "drug* facilitat* sex*" OR (sex* PRE/4 "after inject*") OR "alcohol consum* during sex*" OR "intercours* while intoxicat*" OR (intercours* PRE/4 "under the influence") OR "intercours* while drunk" OR "intercours* while high" OR "intercours* while drink*" OR "sex while drink*" OR "sex* inject* drug*" OR "drug* for sex*" OR "alcohol for sex*" OR "sexual pract* while" OR "sexual pract* under the influence" OR "sexual* substance* us*” OR "substance* during sex*" OR "substance* us* during sex*" OR (substance PRE/2 "before sex*") OR (substance PRE/2 "prior to sex*")) |
| 3 | 1 AND 2 |
| 4 | FILTER: Stype.exact ("Scholarly Journals" or "Dissertations & Theses") |
| 5 | FILTER: La.exact (English or Spanish) |
| 6 | FILTER: Yr (2000-2029) |

**Supplementary Table S2c**

*Scopus search strategy*

| Step Search terms | |
| --- | --- |
| 1 | ( ( TITLE-ABS-KEY ( youth* ) OR TITLE-ABS-KEY ( young* ) OR TITLE-ABS-KEY ( student* ) OR TITLE-ABS-KEY ( "young adult*" ) OR TITLE-ABS-KEY ( juvenil* ) OR TITLE-ABS-KEY ( "emerging adult*" ) OR TITLE-ABS-KEY ( "young people" ) OR TITLE-ABS-KEY ( "young student*" ) OR TITLE-ABS-KEY ( "university student*" ) OR TITLE-ABS-KEY ( universit* ) OR TITLE-ABS-KEY ( college ) OR TITLE-ABS-KEY ( undergraduat* ) OR TITLE-ABS-KEY ( postgraduat* ) OR TITLE-ABS-KEY ( graduat* ) OR TITLE-ABS-KEY ( postsecondary ) OR TITLE-ABS-KEY ( "higher education" ) OR TITLE-ABS-KEY ( "tertiary education" ) OR TITLE-ABS-KEY ( bachelor* ) OR TITLE-ABS-KEY ( "master* degree*" ) OR TITLE-ABS-KEY ( "doctoral degree" ) OR TITLE-ABS-KEY ( freshman ) OR TITLE-ABS-KEY ( "early adult*" ) OR TITLE-ABS-KEY ( "adulthood" ) OR TITLE-ABS-KEY ( "college-age*" ) OR TITLE-ABS-KEY ( "late adolescen*" ) OR TITLE-ABS-KEY ( "older adolescen*" ) OR TITLE-ABS-KEY ( "junior college" ) OR TITLE-ABS-KEY ( "college senior*" ) OR TITLE-ABS-KEY ( "18 to 29" ) OR TITLE-ABS-KEY ( "18-29" ) ) ) |
| 2 | ( ( TITLE-ABS-KEY ( chemsex ) OR TITLE-ABS-KEY ( "sex* drug* use" ) OR TITLE-ABS-KEY ( "sex* drug* tak*" ) OR TITLE-ABS-KEY ( "drug* facilitat* sex*" ) OR TITLE-ABS-KEY ( "alcohol facilitat* sex*" ) OR TITLE-ABS-KEY ( "drug* enhanc* sex*" ) OR TITLE-ABS-KEY ( "alcohol enhanc* sex*" ) OR TITLE-ABS-KEY ( "consum* before sex*" ) OR TITLE-ABS-KEY ( "drug* before sex*" ) OR TITLE-ABS-KEY ( "drug* use before sex*" ) OR TITLE-ABS-KEY ( "alcohol use before sex*" ) OR TITLE-ABS-KEY ( "alcohol before sex*" ) OR TITLE-ABS-KEY ( "sex after consum*" ) OR TITLE-ABS-KEY ( "sex under the influence" ) OR TITLE-ABS-KEY ( "sex while us* drug*" ) OR TITLE-ABS-KEY ( "sex* while under the influence" ) OR TITLE-ABS-KEY ( "sex while drunk*" ) OR TITLE-ABS-KEY ( "sex while high" ) OR TITLE-ABS-KEY ( "drug* prior to sex*" ) OR TITLE-ABS-KEY ( "alcohol prior to sex*" ) OR TITLE-ABS-KEY ( "consum* prior to sex*" ) OR TITLE-ABS-KEY ( "sex after inject*" ) OR TITLE-ABS-KEY ( "sex while intoxicat*" ) OR TITLE-ABS-KEY ( "drug* during sex*" ) OR TITLE-ABS-KEY ( "alcohol during sex*" ) OR TITLE-ABS-KEY ( "alcohol use during sex*" ) OR TITLE-ABS-KEY ( "alcohol consum* during sex*" ) OR TITLE-ABS-KEY ( "drug* use during sex*" ) OR TITLE-ABS-KEY ( "intak* before sex*" ) OR TITLE-ABS-KEY ( "intak* during sex*" ) OR TITLE-ABS-KEY ( slamming ) OR TITLE-ABS-KEY ( slamsex ) OR TITLE-ABS-KEY ( "sex* inject* drug use" ) OR TITLE-ABS-KEY ( "inject* during sex*" ) OR TITLE-ABS-KEY ( "inject* sex* drug*" ) OR TITLE-ABS-KEY ( "Party-N-Play" ) OR TITLE-ABS-KEY ( "Party and Play" ) OR TITLE-ABS-KEY ( "sex* after us*" ) OR TITLE-ABS-KEY ( "intercourse while intoxicat*" ) OR TITLE-ABS-KEY ( "intercourse under the influence" ) OR TITLE-ABS-KEY ( "intercourse while drunk*" ) OR TITLE-ABS-KEY ( "intercourse while high" ) OR TITLE-ABS-KEY ( "intercourse while under the influence" ) OR TITLE-ABS-KEY ( "intercourse while drink*" ) OR TITLE-ABS-KEY ( "sex while drink*" ) OR TITLE-ABS-KEY ( "sex* inject* drug*" ) OR TITLE-ABS-KEY ( "drug* for sex" ) OR TITLE-ABS-KEY ( "alcohol for sex*" ) OR TITLE-ABS-KEY ( "sexual practic* while intoxicat*" ) OR TITLE-ABS-KEY ( "sexual practic* under the influence" ) OR TITLE-ABS-KEY ( "sexual practic* while under the influence" ) OR TITLE-ABS-KEY ( "drug* us* prior to sex*" ) OR TITLE-ABS-KEY ( "alcohol us* prior to sex*" ) OR TITLE-ABS-KEY ( " sexual* substanc* us* " ) OR TITLE-ABS-KEY ( " substanc* during sex* " ) OR TITLE-ABS-KEY ( " substanc* us* at last sex " ) OR TITLE-ABS-KEY ( " substanc* us* before sex* " ) OR TITLE-ABS-KEY ( " substanc* us* prior to sex* " ) ) ) |
| 3 | 1 AND 2 |
| 4 | FILTER: Publication Year (2000-2020) |
| 5 | FILTER: Document Type- excluded ("cp" or "ch" or “no” or “cr” or “er” or “le”) |
| 6 | FILTER: Language (English or Spanish) |

**Supplementary Table S2d**

*Grey literature search strategy*

| Search characteristics | | Research descriptors and expressions | |
| --- | --- | --- | --- |
|  |  | Exposure | Population |
| Sources | Google scholar and Google | Chemsex | Young adults |
| Where the words occur | Anywhere in the publication | Sexualized drug use | Emerging adults |
| Sorted by | Relevance | Sex under the influence | Youths |
| Published between | January 2000 to October 2021 | Use before sex | University students |
| Reviewed the first | 150 results of each year | Sex while under the influence of |  |
| Limits applied | No | Sex while high |  |
| Alerts activated | No | Sex while intoxicated |  |
|  |  | Sex after consumption |  |
