# Supplementary material for: Prevalence of Intoxicating Substance Use Before or During Sex Among Young Adults: A Systematic Review and Meta-Analysis
Source: Arch Sex Behav. 2023 Mar 10;52(6):2503–26. doi: 10.1007/s10508-023-02572-z (PMC10501956; doi:10.1007/s10508-023-02572-z)
Supplement: Supplementary file 2 — Supplementary file2 (DOCX 52 KB) [file 10508_2023_2572_MOESM2_ESM.docx]

**Supplementary Table S2**

*Reasons why studies examined on the basis of their full text were excluded and their references*

| **Nº** | **Study** | **Assessing prevalence data** | **Prevalence data** | **Demographic or methodological description** | **Other** |
| --- | --- | --- | --- | --- | --- |
| 1 | Abdullah et al. (2003) | - | - | - | Does not meet our age criteria |
| 2 | Bailey et al. (2012) | - | Not calculable | - | - |
| 3 | Bauer et al. (2002) | - | - | - | Does not meet our age criteria |
| 4 | Black et al. (2013) | - | - | - | Does not meet our age criteria |
| 5 | Blanchard et al. (2018) | - | Not reported | - | - |
| 6 | Bond et al. (2002) | - | - | - | Does not meet our age criteria |
| 7 | Bowring et al. (2014) | - | - | Insufficient (sociodemographic) | - |
| 8 | Brooks et al. (2009) | - | Not reported | - | - |
| 9 | Bruce et al. (2013) | - | - | - | Does not meet our age criteria |
| 10 | Bulduk & Erdogan (2012) | - | Not reported | - | - |
| 11 | Buttram & Kurtz (2015) | - | - | - | Does not meet our age criteria |
| 12 | Buttram et al. (2018) | - | - | - | Does not meet our age criteria |
| 13 | Buttram et al. (2019) | - | - | - | Does not meet our age criteria |
| 14 | Caballero et al. (2011) | - | - | - | Does not meet our age criteria |
| 15 | Castro & Santos-Iglesias (2016) | - | Not calculable | - | - |
| 16 | Chen et al. (2019) | - | - | - | Does not meet our age criteria |
| 17 | Chiasson et al. (2007) | - | - | - | Does not meet our age criteria |
| 18 | Clatts et al. (2007) | - | - | - | Does not meet our age criteria |
| 19 | Conner & Flesh (2001) | Was not its goal | - | - | - |
| 20 | Contreras-Landgrave et al. (2020) | - | Inconsistent results | - | - |
| 21 | Costa & Brody (2010) | - | - | - | Does not meet our age criteria |
| 22 | Crosby (2019) | - | - | Insufficient (both) | - |
| 23 | Curry et al. (2018) | - | Not reported | - | - |
| 24 | Danube et al. (2014) | Was not its goal | - | - | - |
| 25 | Donohew et al. (2000) | - | Not reported | - | - |
| 26 | Dunn et al. (2017) | - | Not reported | - | - |
| 27 | Feeney et al. (2000) | Was not its goal | - | - | - |
| 28 | Fernández et al. (2005) | - | - | - | Does not meet our age criteria |
| 29 | Fierros-Gonzalez & Brown (2002) | - | - | - | Does not meet our age criteria |
| 30 | Fisher et al. (2010) | - | - | - | Does not meet our age criteria |
| 31 | Folch et al. (2006) | - | - | - | Does not meet our age criteria |
| 32 | Garga et al. (2021) | - | - | - | Does not meet our age criteria |
| 33 | Gause et al. (2019) | - | - | Inconsistent age range description | - |
| 34 | Gerbi et al. (2009) | - | - | - | Does not meet our age criteria |
| 35 | Giles et al. (2009) | - | - | - | Does not meet our age criteria |
| 36 | Haugstvedt et al. (2018) | - | - | - | Norwegian language |
| 37 | Hendershot et al. (2007) | - | - | - | Does not meet our age criteria |
| 38 | Herrick et al. (2013) | - | - | - | Does not meet our age criteria |
| 39 | Hogben et al. (2001) | - | - | - | Does not meet our age criteria |
| 40 | Holmes (2008) | - | - | - | Does not meet our age criteria |
| 41 | Hooshyar et al. (2018) | - | Inconsistent results | - | - |
| 42 | Hou (2007) | - | Not calculable | - | - |
| 43 | Hou (2009) | - | Not reported | - | - |
| 44 | Huibregtse et al. (2021) | - | Not calculable | - | - |
| 45 | Jozkowski & Wiersma (2015) | - | - | - | Does not meet our age criteria |
| 46 | Kahumoku-Fessler (2011) | - | Not reported | - | - |
| 47 | Kebede et al. (2018) | Was not its goal | - | - | - |
| 48 | Kiene et al. (2008) | - | - | - | Does not meet our age criteria |
| 49 | Koenig et al. (2004) | - | - | - | Does not meet our age criteria |
| 50 | Lanza-Kaduce et al. (2006) | - | - | Insufficient (sociodemographic) | - |
| 51 | Lelutiu-Weinberger et al. (2015) | - | Not reported | - | - |
| 52 | Li et al. (2014) | - | Not reported | - | - |
| 53 | Litovich (2009) | - | Not reported | - | - |
| 54 | Logan & Leukefeld (2000) | - | - | - | Does not meet our age criteria |
| 55 | Lomba et al. (2009) | - | - | - | Does not meet our age criteria |
| 56 | Macapagal et al. (2016) | - | - | - | Does not meet our age criteria |
| 57 | MacGowan et al. (2003) | - | Not reported | - | - |
| 58 | Madhivanan et al. (2005) | - | - | - | Does not meet our age criteria |
| 59 | Maksut et al. (2016) | - | Not reported | - | - |
| 60 | Manhart et al. (2016) | - | Not calculable | - | - |
| 61 | Marcantonio & Jozkowski (2021) | Was not its goal | - | - | - |
| 62 | Meade et al. (2009) | - | - | - | Does not meet our age criteria |
| 63 | Messman-Moore et al. (2013) | - | - | - | Does not meet our age criteria |
| 64 | Moore & Davidson (2006) | Was not its goal | - | - | - |
| 65 | Moure-Rodríguez et al. (2016) | - | - | - | Does not meet our age criteria |
| 66 | Munroe et al. (2010) | - | - | - | Does not meet our age criteria |
| 67 | Mwaba (2009) | - | - | Insufficient (both) | - |
| 68 | Nagayama et al. (2000) | - | - | Insufficient (sociodemographic) | - |
| 69 | Nakamura (2007) | - | - | - | Does not meet our age criteria |
| 70 | Nemoto et al. (2004) | - | - | - | Does not meet our age criteria |
| 71 | NIMH Multisite HIV Prevention Trial Group (2001) | - | - | - | Does not meet our age criteria |
| 72 | Norona et al. (2021) | - | Not reported | - | - |
| 73 | Nugent et al. (2010) | - | - | - | Does not meet our age criteria |
| 74 | Operario & Nemoto (2005) | - | - | - | Does not meet our age criteria |
| 75 | Paat et al. (2021) | - | Not reported | - | - |
| 76 | Parkhill (2006) | - | Not reported | - | - |
| 77 | Parsons et al. (2004) | - | - | - | Does not meet our age criteria |
| 78 | Peasant et al. (2019) | - | Not calculable | - | - |
| 79 | Pechansky et al. (2011) | - | - | - | Does not meet our age criteria |
| 80 | Poppen et al. (2004) | - | - | - | Does not meet our age criteria |
| 81 | Potard (2020) | - | - | - | Does not meet our age criteria |
| 82 | Radcliffe et al. (2010) | - | - | - | Does not meet our age criteria |
| 83 | Randolph & Mosack (2006) | - | - | - | Does not meet our age criteria |
| 84 | Reback et al. (2005) | - | - | - | Does not meet our age criteria |
| 85 | Reid (2013) | - | Not calculable | - | - |
| 86 | Reynolds et al. (2008) | - | - | - | Does not meet our age criteria |
| 87 | Rickert et al. (2000) | - | - | Insufficient (both) | - |
| 88 | Rios-González et al. (2018) | - | - | - | Does not meet our age criteria |
| 89 | Rosińska et al. (2016) | - | - | Insufficient (sociodemographic) | - |
| 90 | Sabato (2016) | - | Not calculable | - | - |
| 91 | Sekirime et al. (2001) | - | - | Insufficient (sociodemographic) | - |
| 92 | Sheehan et al. (2016) | Was not its goal | - | - | - |
| 93 | Simons et al. (2005) | - | - | - | Does not meet our age criteria |
| 94 | Solomon et al. (2011) | Was not its goal | - | - | - |
| 95 | Ssewanyana et al. (2015) | - | Not reported | - | - |
| 96 | Stanton et al. (2001) | Was not its goal | - | - | - |
| 97 | Strandberg et al. (2019) | - | - | - | Does not meet our age criteria |
| 98 | Tan et al. (2021) | - | Duplicated sample | - | - |
| 99 | Thompson et al. (2014) | - | Not reported | - | - |
| 100 | Thompson et al. (2020) | - | Not reported | - | - |
| 101 | Tolou-Shams et al. (2007) | - | - | - | Does not meet our age criteria |
| 102 | Tolou-Shams et al. (2008) | - | - | - | Does not meet our age criteria |
| 103 | Wechsberg et al. (2008) | - | - | - | Does not meet our age criteria |
| 104 | Wells et al. (2010) | - | - | - | Sample with alcohol consumption patterns |
| 105 | Wesche et al. (2021) | - | Not calculable | - | - |
| 106 | Wheater et al. (2003) | - | - | Insufficient (sociodemographic) | - |
| 107 | Wilton (2008) | - | - | - | Does not meet our age criteria |
| 108 | Young et al. (2017) | - | - | - | Does not meet our age criteria |
| 109 | Zullig et al. (2010) | Was not its goal | - | - | - |

Note: The total of 109 studies includes 103 studies identified via the databases and 6 non-duplicated studies identified via other sources.

References of excluded studies examined based on full-text:

Abdullah, A. S. M., Fielding, R., & Hedley, A. J. (2003). Understanding sexual risk-taking behaviour in Hong Kong university students: A health promotion perspective. *Preventive Medicine*, *37*(4), 311–318. https://doi.org/10.1016/S0091-7435(03)00138-5

Bailey, J. A., Fleming, C. B., Catalano, R. F., Haggerty, K. P., & Manhart, L. E. (2012). Romantic relationship characteristics and alcohol use: Longitudinal associations with dual method contraception use. *Journal of Adolescent Health*, *50*(5), 450–455. https://doi.org/10.1016/j.jadohealth.2011.09.008

Bauer, H. M., Gibson, P., Hernandez, M., Kent, C., Klausner, J., & Bolan, G. (2002). Intimate partner violence and high-risk sexual behaviors among female patients with sexually transmitted diseases. *Sexually Transmitted Diseases*, *29*(7), 411–416. https://doi.org/10.1097/00007435-200207000-00009

Black, S. R., Schmiege, S., & Bull, S. (2013). Actual versus perceived peer sexual risk behavior in online youth social networks. *Translational Behavioral Medicine*, *3*(3), 312–319. https://doi.org/10.1007/s13142-013-0227-y

Blanchard, B. E., Stevens, A. K., Acosta, I. S., Talley, A. E., Brown, J. L., & Littlefield, A. K. (2018). The influence of motives on alcohol- and sex-related behaviors among female college students. *Drug and Alcohol Dependence*, *185*, 120–126. https://doi.org/10.1016/j.drugalcdep.2017.11.039

Bond, L., Lavelle, K., & Lauby, J. (2002). A comparison of the risk characteristics of ever-pregnant and never-pregnant sexually active adolescents. *Journal of HIV/AIDS Prevention & Education for Adolescents & Children*, *5*(1–2), 123–137. https://doi.org/10.1300/J129v05n01_08

Bowring, A. L., Van Gemert, C., Vongsaiya, K., Hughes, C., Sihavong, A., Phimphachanh, C., Chanlivong, N., Agius, P. A., Toole, M., & Hellard, M. (2014). Setting the scene: Locations for meeting sex partners among behaviorally bisexual men in Vientiane, Laos, and opportunities for health promotion. *AIDS Education and Prevention*, *26*(6), 538–553. https://doi.org/10.1521/aeap.2014.26.6.538

Brooks, R. A., Lee, S. J., Stover, G. N., & Barkley, T. W. (2009). Condom attitudes, perceived vulnerability, and sexual risk behaviors of young Latino male urban street gang members: Implications for HIV prevention. *AIDS Education and Prevention*, *21*(5 Suppl.), 80–87. https://doi.org/10.1521/aeap.2009.21.5-supp.80

Bruce, D., Kahana, S., Harper, G. W., & Fernández, M. I. (2013). Alcohol use predicts sexual risk behavior with HIV-negative or partners of unknown status among young HIV-positive men who have sex with men. *AIDS Care - Psychological and Socio-Medical Aspects of AIDS/HIV*, *25*(5), 559–565. https://doi.org/10.1080/09540121.2012.720363

Bulduk, S., & Erdogan, S. (2012). The effects of peer education on reduction of the HIV/sexually transmitted infection risk behaviors among Turkish university students. *Journal of the Association of Nurses in AIDS Care*, *23*(3), 233–243. https://doi.org/10.1016/j.jana.2011.02.003

Buttram, M. E., & Kurtz, S. P. (2015). Characteristics associated with group sex participation among men and women in the club drug scene. *Sexual Health*, *12*(6), 560–562. https://doi.org/10.1071/SH15071

Buttram, M. E., Pagano, M. E., & Kurtz, S. P. (2018). Frequency of group sex participation and risk for HIV/STI among young adult nightclub scene participants. *International Journal of Sexual Health*, *30*(1), 12–19. https://doi.org/10.1080/19317611.2017.1385561

Buttram, M. E., Pagano, M. E., & Kurtz, S. P. (2019). Foster care, syndemic health disparities and associations with HIV/STI diagnoses among young adult substance users. *Sexually Transmitted Infections*, *95*(3), 175–180. https://doi.org/10.1136/sextrans-2017-053490

Caballero Badillo, M. C., Camargo Figuera, F. A., & Castro, B. Z. (2011). Prácticas inadecuadas del uso del condón y factores asociados en estudiantes universitarios. *Revista de La Universidad Industrial de Santander. Salud*, *43*(3), 257–262.

Castro, Á., & Santos-Iglesias, P. (2016). Sexual behavior and sexual risks among Spanish university students: A descriptive study of gender and sexual orientation. *Sexuality Research and Social Policy*, *13*, 84–94. https://doi.org/10.1007/s13178-015-0210-0

Chen, Y. T., Kolak, M., Duncan, D. T., Schumm, P., Michaels, S., Fujimoto, K., & Schneider, J. A. (2019). Neighbourhoods, networks and pre-exposure prophylaxis awareness: A multilevel analysis of a sample of young black men who have sex with men. *Sexually Transmitted Infections*, *95*(3), 228–235. https://doi.org/10.1136/sextrans-2018-053639

Chiasson, M. A., Hirshfield, S., Remien, R. H., Humberstone, M., Wong, T., & Wolitski, R. J. (2007). A comparison of on-line and off-line sexual risk in men who have sex with men: An event-based on-line survey. *Journal of Acquired Immune Deficiency Syndromes*, *44*(2), 235–243. https://doi.org/10.1097/QAI.0b013e31802e298c

Clatts, M. C., Giang, L. M., Goldsamt, L. A., & Yi, H. (2007). Male sex work and HIV risk among young heroin users in Hanoi, Vietnam. *Sexual Health*, *4*(4), 261–267. https://doi.org/10.1071/SH07018

Conner, M., & Flesch, D. (2001). Having casual sex: Additive and interactive effects of alcohol and condom availability on the determinants of intentions. *Journal of Applied Social Psychology*, *31*, 89–112. https://doi.org/10.1111/j.1559-1816.2001.tb02484.x

Contreras-Landgrave, G., Ibarra-Espinosa, M. L., Casas-Patiño, Ó. D., Camacho-Ruiz, E. J., & Velasco-Cañas, D. F. (2020). El conocimiento sobre métodos anticonceptivos y la conducta sexual en jóvenes universitarios. *Hacia La Promoción de La Salud*, *25*(2), 70–83. https://doi.org/10.17151/hpsal.2020.25.2.9

Costa, R. M., & Brody, S. (2010). Immature defense mechanisms are associated with lesser vaginal orgasm consistency and greater alcohol consumption before sex. *Journal of Sexual Medicine*, *7*, 775–786. https://doi.org/10.1111/j.1743-6109.2009.01559.x

Crosby, R. A. (2019). Alcohol exacerbates the association between partner-related issues regarding condom use and condomless anal receptive sex among young black men who have sex with men. *Sexual Health*, *16*(6), 600–601. <https://doi.org/10.1071/SH19030>

Curry, I., Luk, J. W., Trim, R. S., Hopfer, C. J., Hewitt, J. K., Stallings, M. C., Brown, S. A., & Wall, T. L. (2018). Impulsivity dimensions and risky sex behaviors in an at-risk young adult sample. *Archives of Sexual Behavior*, *47*, 529–536. https://doi.org/10.1007/s10508-017-1054-x

Danube, C. L., Vescio, T. K., & Davis, K. C. (2014). Male role norm endorsement and sexism predict heterosexual college men’s attitudes toward casual sex, intoxicated sexual contact, and casual sex. *Sex Roles*, *71*(5–8), 219–232. https://doi.org/10.1007/s11199-014-0394-4

Donohew, L., Zimmerman, R., Cupp, P. S., Novak, S., Colon, S., & Abell, R. (2000). Sensation seeking, impulsive decision-making, and risky sex: Implications for risk-taking and design of interventions. *Personality and Individual Differences*, *28*(6), 1079–1091. https://doi.org/10.1016/S0191-8869(99)00158-0

Dunn, M. E., Borjesson-Holman, W., & Tantleff-Dunn, S. (2017). Sexual misconduct on campus: Compliance problems with a sexual conduct code for college students and potential solutions. *The Open Psychology Journal*, *10*(1), 170–181. https://doi.org/10.2174/1874350101710010170

Feeney, J. A., Peterson, C., Gallois, C., & Terry, D. J. (2000). Attachment style as a predictor of sexual attitudes and behavior in late adolescence. *Psychology and Health*, *14*(6), 1105–1122. https://doi.org/10.1080/08870440008407370

Fernández, M. I., Bowen, G. S., Varga, L. M., Collazo, J. B., Hernandez, N., Perrino, T., & Rehbein, A. (2005). High rates of club drug use and risky sexual practices among Hispanic men who have sex with men in Miami, Florida. *Substance Use & Misuse*, *40*, 1347–1362. https://doi.org/10.1081/JA-200066904

Fierros-Gonzalez, R., & Brown, J. M. (2002). High risk behaviors in a sample of Mexican-American college students. *Psychological Reports*, *90*, 117–130. https://doi.org/10.2466/pr0.2002.90.1.117

Fisher, J. C., Cook, P. A., & Kapiga, S. H. (2010). Alcohol use before sex and HIV risk: Situational characteristics of protected and unprotected encounters among high-risk African women. *Sexually Transmitted Diseases*, *37*(9), 571–578. https://doi.org/10.1097/OLQ.0b013e3181dbafad

Folch, C., Marks, G., Esteve, A., Zaragoza, K., Muñoz, R., & Casabona, J. (2006). Factors associated with unprotected sexual intercourse with steady male, casual male, and female partners among men who have sex with men in Barcelona, Spain. *AIDS Education and Prevention*, *18*(3), 227–242. https://doi.org/10.1521/aeap.2006.18.3.227

Garga, S., Thomas, M., Bhatia, A., Sullivan, A., John-Leader, F., & Pit, S. (2021). Geosocial networking dating app usage and risky sexual behavior in young adults attending a music festival: Cross-sectional questionnaire study. *Journal of Medical Internet Research*, *23*(4): e21082. https://doi.org/10.2196/21082

Gause, N. K., Brown, J. L., & DiClemente, R. J. (2019). Mental representation of self in relationships indirectly affects young black women’s engagement in risky sexual behaviors through psychosocial HIV/STI risk factors. *Vulnerable Children and Youth Studies*, *14*(1), 1–16. https://doi.org/10.1080/17450128.2019.1574366

Gerbi, G. B., Habtemariam, T., Tameru, B., Nganwa, D., & Robnett, V. (2009). The correlation between alcohol consumption and risky sexual behaviours among people living with HIV/AIDS. *Journal of Substance Use*, *14*(2), 90–100. https://doi.org/10.1080/14659890802624261

Giles, S. M., Champion, H., Sutfin, E. L., McCoy, T. P., & Wagoner, K. (2009). Calorie restriction on drinking days: An examination of drinking consequences among college students. *Journal of American College Health*, *57*(6), 603–610. https://doi.org/10.3200/JACH.57.6.603-610

Haugstvedt, Å., Amundsen, E., & Berg, R. C. (2018). Chemsex blant menn - en spørreundersøkelse. *Tidsskrift for Den Norske Laegeforening*, *138*(13), 1–9. <https://doi.org/10.4045/tidsskr.18.0108>

Hendershot, C. S., Stoner, S. A., George, W. H., & Norris, J. (2007). Alcohol use, expectancies, and sexual sensation seeking as correlates of HIV risk behavior in heterosexual young adults. *Psychology of Addictive Behaviors*, *21*(3), 365–372. https://doi.org/10.1037/0893-164X.21.3.365

Herrick, A., Kuhns, L., Kinsky, S., Johnson, A., & Garofalo, R. (2013). Demographic, psychosocial, and contextual factors associated with sexual risk behaviors among young sexual minority women. *Journal of the American Psychiatric Nurses Association*, *19*(6), 345–355. https://doi.org/10.1177/1078390313511328

Hogben, M., St. Lawrence, J., & Eldridge, G. D. (2001). Sexual risk behavior, drug use, and STD rates among incarcerated women. *Women and Health*, *34*(1), 63–78. https://doi.org/10.1300/J013v34n01_05

Holmes, W. C. (2008). Men’s self-definitions of abusive childhood sexual experiences, and potentially related risky behavioral and psychiatric outcomes. *Child Abuse and Neglect*, *32*(1), 83–97. https://doi.org/10.1016/j.chiabu.2007.09.005

Hooshyar, S. H., Karamouzian, M., Mirzazadeh, A., Haghdoost, A. A., Sharifi, H., & Shokoohi, M. (2018). Condom use and its associated factors among Iranian youth: Results from a population-based study. *International Journal of Health Policy and Management*, *7*(11), 1007–1014. https://doi.org/10.15171/ijhpm.2018.65

Hou, S. I. (2007). Alternative modes of measuring self-reports on HIV-related behaviors among college students: Web-delivered mode versus paper-pencil mode. *American Journal of Health Education*, *38*(1), 9–15. https://doi.org/10.1080/19325037.2007.10598937

Hou, S. I. (2009). HIV-related behaviors among black students attending historically black colleges and universities (HBCUs) versus white students attending a traditionally white institution (TWI). *AIDS Care - Psychological and Socio-Medical Aspects of AIDS/HIV*, *21*(8), 1050–1057. https://doi.org/10.1080/09540120802626196

Huibregtse, B. M., Hatoum, A. S., Corley, R. P., Rhea, S. A., Hewitt, J. K., & Stallings, M. C. (2021). Etiological overlap between sex under the influence and number of lifetime sexual partners. *Behavior Genetics*, *51*(1), 12–29. https://doi.org/10.1007/s10519-020-10019-7

Jozkowski, K. N., & Wiersma, J. D. (2015). Does drinking alcohol prior to sexual activity influence college students’ consent? *International Journal of Sexual Health*, *27*(2), 156–174. https://doi.org/10.1080/19317611.2014.951505

Kahumoku-Fessler, E. P. (2011). *Links between body image and internalizing behaviors among college-age youth: Similarities or differences by sex, level of acculturation, and racial/ethnic groups, and, who's “doing” what?: An investigation of regional trends in sexual activity and risky sexual behaviors among college-age youth* [Doctoral dissertation, Auburn University]. ProQuest Dissertations and Theses. search.proquest.com/docview/923286772?accountid=14553%5Cnhttp://openurl.library.uiuc.edu/sfxlcl3?url_ver=Z39.882004&rft_val_fmt=info:ofi/fmt:kev:mtx:dissertation&genre=dissertations+&+theses&sid=ProQ:ProQuest+Dissertations+&+Theses+Full+Text&atitl

Kebede, A., Molla, B., & Gerensea, H. (2018). Assessment of risky sexual behavior and practice among Aksum university students, Shire Campus, Shire Town, Tigray, Ethiopia, 2017. *BMC Research Notes*, *11*(1), 88. https://doi.org/10.1186/s13104-018-3199-7

Kiene, S. M., Simbayi, L. C., Abrams, A., Cloete, A., Tennen, H., & Fisher, J. D. (2008). High rates of unprotected sex occurring among HIV-positive individuals in a daily diary study in South Africa: The role of alcohol use. *Journal of Acquired Immune Deficiency Syndromes*, *49*(2), 219–226. https://doi.org/10.1097/QAI.0b013e318184559f

Koenig, M. A., Lutalo, T., Zhao, F., Nalugoda, F., Kiwanuka, N., Wabwire-Mangen, F., Kigozi, G., Sewankambo, N., Wagman, J., Serwadda, D., Wawer, M., & Gray, R. (2004). Coercive sex in rural Uganda: Prevalence and associated risk factors. *Social Science and Medicine*, *58*(4), 787–798. https://doi.org/10.1016/S0277-9536(03)00244-2

Lanza-Kaduce, L., Capece, M., & Alden, H. (2006). Liquor is quicker: Gender and social learning among college students. *Criminal Justice Policy Review*, *17*(2), 127–143. https://doi.org/10.1177/0887403405279934

Lelutiu-Weinberger, C., Pachankis, J. E., Gamarel, K. E., Surace, A., Golub, S. A., & Parsons, J. T. (2015). Feasibility, acceptability, and preliminary efficacy of a live-chat social media intervention to reduce HIV risk among young men who have sex with men. *AIDS and Behavior*, *19*(7), 1214–1227. https://doi.org/10.1007/s10461-014-0911-z

Li, M. J., Distefano, A., Mouttapa, M., & Gill, J. K. (2014). Bias-motivated bullying and psychosocial problems: Implications for HIV risk behaviors among young men who have sex with men. *AIDS Care - Psychological and Socio-Medical Aspects of AIDS/HIV*, *26*(2), 246–256. https://doi.org/10.1080/09540121.2013.811210

Litovich, M. L. (2009). *Involvement in gay men’s communities and HIV-related sexual risk behaviour* [Doctoral dissertation, University of Maryland, Baltimore Country]. ProQuest Dissertations & Theses Global. https://www.proquest.com/dissertations-theses/involvement-gay-mens-communities-hiv-related/docview/288079502/se-2

Logan, T. K., & Leukefeld, C. (2000). Sexual and drug use behaviors among female crack users: A multi-site sample. *Drug and Alcohol Dependence*, *58*(3), 237–245. https://doi.org/10.1016/S0376-8716(99)00096-4

Lomba, L., Apóstolo, J., & Mendes, F. (2009). Drugs and alcohol consumption and sexual behaviours in night recreational settings in Portugal. *Adicciones*, *21*(4), 309–326. https://doi.org/10.20882/adicciones.222

Macapagal, K., Greene, G. J., Andrews, R., & Mustanski, B. (2016). Evaluating the relationship-oriented information, motivation, and behavioral skills model of HIV preventive behaviors in young men who have sex with men. *AIDS Education and Prevention*, *28*(2), 165–179. https://doi.org/10.1521/aeap.2016.28.2.165

MacGowan, R. J., Margolis, A., Gaiter, J., Morrow, K., Zack, B., Askew, J., McAuliffe, T., Sosman, J. M., & Eldridge, G. D. (2003). Predictors of risky sex of young men after release from prison. *International Journal of STD & AIDS*, *14*(8), 519–523. https://doi.org/10.1258/095646203767869110

Madhivanan, P., Hernandez, A., Gogate, A., Stein, E., Gregorich, S., Setia, M., Kumta, S., Ekstrand, M., Mathur, M., Jerajani, H., & Lindan, C. P. (2005). Alcohol use by men is a risk factor for the acquisition of sexually transmitted infections and human immunodeficiency virus from female sex workers in Mumbai, India. *Sexually Transmitted Diseases*, *32*(11), 685–690. https://doi.org/10.1097/01.olq.0000175405.36124.3b

Maksut, J. L., Eaton, L. A., Siembida, E. J., Driffin, D. D., & Baldwin, R. (2016). An evaluation of factors associated with sexual risk taking among black men who have sex with men: A comparison of younger and older populations. *Journal of Behavioral Medicine*, *39*(4), 665–674. https://doi.org/10.1007/s10865-016-9734-x

Manhart, L. E., Epstein, M., Bailey, J. A., Hill, K. G., Haggerty, K. P., & Catalano, R. F. (2016). HIV/Sexually transmitted infection prevention messaging: targeting root causes of sexual risk behavior. *Sexually Transmitted Diseases*, *43*(2), 71–77. https://doi.org/10.1097/OLQ.0000000000000402

Marcantonio, T. L., & Jozkowski, K. N. (2021). Do college students feel confident to consent to sex after consuming alcohol? *Journal of American College Health*. https://doi.org/10.1080/07448481.2021.1943413

Meade, C. S., McDonald, L. J., & Weiss, R. D. (2009). HIV risk behavior in opioid dependent adults seeking detoxification treatment: An exploratory comparison of heroin and oxycodone users. *American Journal on Addictions*, *18*(4), 289–293. https://doi.org/10.1080/10550490902925821

Messman-Moore, T. L., Ward, R. M., & DeNardi, K. A. (2013). The impact of sexual enhancement alcohol expectancies and risky behavior on alcohol-involved rape among college women. *Violence Against Women*, *19*(4), 449–464. https://doi.org/10.1177/1077801213487058

Moore, N. B., & Davidson, J. K. (2006). College women and personal goals: Cognitive dimensions that differentiate risk-reduction sexual decisions. *Journal of Youth and Adolescence*, *35*(4), 577–589. <https://doi.org/10.1007/s10964-006-9041-x>

Moure-Rodríguez, L., Doallo, S., Juan-Salvadores, P., Corral, M., Cadaveira, F., & Caamaño-Isorna, F. (2016). Consumo intensivo de alcohol y cannabis, y prácticas sexuales de riesgo en estudiantes universitarios. *Gaceta Sanitaria*, *30*(6), 438–443. https://doi.org/10.1016/j.gaceta.2016.03.007

Munroe, C. D., Kibler, J. L., Ma, M., Dollar, K. M., & Coleman, M. (2010). The relationship between posttraumatic stress symptoms and sexual risk: Examining potential mechanisms. *Psychological Trauma: Theory, Research, Practice, and Policy*, *2*(1), 49–53. https://doi.org/10.1037/a0018960

Mwaba, K. (2009). Attitudes and beliefs about homosexuality and same-sex marriage among a sample of South African students. *Social Behavior and Personality*, *37*(6), 801–804. https://doi.org/10.2224/sbp.2009.37.6.801

Nagayama Hall, G. C., Sue, S., Narang, D. S., & Lilly, R. S. (2000). Culture-specific models of men’s sexual aggression: Intra- and interpersonal determinants. *Cultural Diversity and Ethnic Minority Psychology*, *6*(3), 252–267. https://doi.org/10.1037/1099-9809.6.3.252

Nakamura, N. (2007). *The relationship between social oppression, internalized homonegativity, substance use during sex and risky sexual behavior in Latino gay and bisexual men* [Doctoral dissertation, The George Washington University]. ProQuest Dissertations & Theses Global. http://search.proquest.com/docview/304875121?accountid=25355

Nemoto, T., Operario, D., Keatley, J. A., Han, L., & Soma, T. (2004). HIV risk behaviors among male-to-female transgender persons of color in San Francisco. *American Journal of Public Health*, *94*(7), 1193–1199. https://doi.org/10.2105/AJPH.94.7.1193

NIMH Multisite HIV Prevention Trial Group (2001). A test of factors mediating the relationship between unwanted sexual activity during childhood and risky sexual practices among women enrolled in the NIMH multisite HIV prevention trial. *Women and Health*, *33*(1–2), 163–180. https://doi.org/10.1300/J013v33n01_10

Norona, J. C., Borsari, B., Oesterle, D. W., & Orchowski, L. M. (2021). Alcohol use and risk factors for sexual aggression: Differences according to relationship status. *Journal of Interpersonal Violence*, *36*(9–10), NP5125–NP5147. <https://doi.org/10.1177/0886260518795169>

Nugent, N. R., Brown, L. K., Beizer, M., Harper, G. W., Nachman, S., & Naar-King, S. (2010). Youth living with HIV and problem substance use: Elevated distress is associated with nonadherence and sexual risk. *Journal of the International Association of Physicians in AIDS Care*, *9*(2), 113–115. https://doi.org/10.1177/1545109709357472

Operario, D., & Nemoto, T. (2005). Sexual risk behavior and substance use among a sample of Asian Pacific Islander transgendered women. *AIDS Education and Prevention*, *17*(5), 430–443. https://doi.org/10.1521/aeap.2005.17.5.430

Paat, Y. F., Orezzoli, M. C. E., & Mangadu, T. (2021). Partner violence among female students attending historically black colleges and universities (HBCUS) in emerging adulthood. *Victims and Offenders*, *16*(4), 519–539. https://doi.org/10.1080/15564886.2020.1818152

Parkhill, M. R. (2006). *The addition of alcohol to the confluence model of sexual* *assault perpetration: A comparison across samples* [Doctoral dissertation, Wayne State University]. Dissertation Abstracts International: Section B: The Sciences and Engineering. https://www.proquest.com/dissertations-theses/addition-alcohol-confluence-model-sexual-assault/docview/621578169/se-2?accountid=14777

Parsons, J. T., Vicioso, K. J., Punzalan, J. C., Halkitis, P. N., Kutnick, A., & Velasquez, M. M. (2004). The impact of alcohol use on the sexual scripts of HIV-positive men who have sex with men. *Journal of Sex Research*, *41*(2), 160–172. https://doi.org/10.1080/00224490409552224

Peasant, C., Montanaro, E. M., Kershaw, T. S., Parra, G. R., Weiss, N. H., Meyer, J. P., Murphy, J. G., Ritchwood, T. D., & Sullivan, T. P. (2019). An event-level examination of successful condom negotiation strategies among young women. *Journal of Health Psychology*, *24*(7), 898–908. https://doi.org/10.1177/1359105317690598

Pechansky, F., Remy, L., Surratt, H. L., Kurtz, S. P., Rocha, T. B. M., Von Diemen, L., Bumaguin, D. B., & Inciardi, J. (2011). Age of sexual initiation, psychiatric symptoms, and sexual risk behavior among ecstasy and LSD users in Porto Alegre, Brazil: A preliminary analysis. *Journal of Drug Issues*, *41*(2), 217–232. https://doi.org/10.1177/002204261104100204

Poppen, P. J., Reisen, C. A., Zea, M. C., Bianchi, F. T., & Echeverry, J. J. (2004). Predictors of unprotected anal intercourse among HIV-positive Latino gay and bisexual men. *AIDS and Behavior*, *8*(4), 379–389. https://doi.org/10.1007/s10461-004-7322-5

Potard, C. (2020). Relationships between women’s emotional reaction to first coital experience and subsequent sexual risk-taking behaviour. *European Journal of Contraception and Reproductive Health Care*, *25*(2), 126–132. https://doi.org/10.1080/13625187.2020.1722993

Radcliffe, J., Doty, N., Hawkins, L. A., Gaskins, C. S., Beidas, R., & Rudy, B. J. (2010). Stigma and sexual health risk in HIV-positive African American young men who have sex with men. *AIDS Patient Care and STDs*, *24*(8), 493–499. https://doi.org/10.1089/apc.2010.0020

Randolph, M. E., & Mosack, K. E. (2006). Factors mediating the effects of childhood sexual abuse on risky sexual behavior among college women. *Journal of Psychology and Human Sexuality*, *18*(1), 23–41. https://doi.org/10.1300/J056v18n01_02

Reback, C. J., Lombardi, E. L., Simon, P. A., & Frye, D. M. (2005). HIV seroprevalence and risk behaviors among transgendered women who exchange sex in comparison with those who do not. *Journal of Psychology & Human Sexuality*, *17*(1–2), 5–22. <https://doi.org/10.1300/J056v17n01_02>

Reid, J. J. (2013). *Relations between sexual identity exploration and risky sexual behavior in emerging adulthood* [Doctoral dissertation, Virginia Commonwealth University]. https://scholarscompass.vcu.edu/etd/3015/

Reynolds, G. L., Latimore, A. D., & Fisher, D. G. (2008). Heterosexual anal sex among female drug users: U.S. national compared to local Long Beach, California data. *AIDS and Behavior*, *12*(5), 796–805. https://doi.org/10.1007/s10461-007-9271-2

Rickert, V. I., Neal, W. P., Wiemann, C. M., & Berenson, A. B. (2000). Prevalence and predictors of low sexual assertiveness. *Journal of Pediatric & Adolescent Gynecology*, *13*(2), 88–89. https://doi.org/10.1016/s1083-3188(00)00016-4

Rios-González, C. M., Verón-Mellid, F. G., De Benedictis-Serrano, G. A., Flores-Enríquez, J. F., & Chirino-Caicedo, A. D. (2018). Prácticas de riesgo para transmisión de VIH en estudiantes de medicina de Latinoamérica y el Caribe, 2017. *Memorias Del Instituto de Investigaciones En Ciencias de La Salud*, *16*(2), 49–54. https://doi.org/10.18004/mem.iics/1812-9528/2018.016(02)49-054

Rosińska, M., Simmons, R., Marzec-Bogusławska, A., Janiec, J., & Porter, K. (2016). Relating HIV testing patterns in Poland to risky and protective behaviour. *AIDS Care - Psychological and Socio-Medical Aspects of AIDS/HIV*, *28*(4), 423–431. https://doi.org/10.1080/09540121.2015.1100702

Sabato, T. (2016). Breaking the model minority stereotype: An exploration of Asian American and Pacific Islander student substance abuse. *College Student Journal*, *50*(4), 477–488.

Sekirime, W. K., Tamale, J., Lule, J. C., & Wabwire-Mangen, F. (2001). Knowledge, attitude and practice about sexually transmitted diseases among university students in Kampala. *African Health Sciences*, *1*(1), 16–22. https://doi.org/10.4314/ahs.v1i1.6822

Sheehan, D. M., Dillon, F. R., Babino, R., Melton, J., Spadola, C., Da Silva, N., & De La Rosa, M. (2016). Recruiting and assessing recent young adult Latina immigrants in health disparities research. *Journal of Multicultural Counseling and Development*, *44*(4), 245–262. https://doi.org/10.1002/jmcd.12052

Simons, L., Lantz, V., Klichine, S., & Ascolese, L. (2005). Drinking games, binge drinking and risky sexual behaviors among college students. *Journal of Alcohol and Drug Education*, *49*(3), 23–26.

Solomon, T. M., Halkitis, P. N., Moeller, R. M., Siconolfi, D. E., Kiang, M. V., & Barton, S. C. (2011). Sex parties among young gay, bisexual, and other men who have sex with men in New York City: Attendance and behavior. *Journal of Urban Health*, *88*(6), 1063–1075. https://doi.org/10.1007/s11524-011-9590-5

Ssewanyana, D., Sebena, R., Petkeviciene, J., Lukács, A., Miovsky, M., & Stock, C. (2015). Condom use in the context of romantic relationships: A study among university students from 12 universities in four Central and Eastern European countries. *European Journal of Contraception and Reproductive Health Care*, *20*(5), 350–360. https://doi.org/10.3109/13625187.2014.1001024

Stanton, B., Li, X., Cottrell, L., & Kaljee, L. (2001). Early initiation of sex, drug-related risk behaviors, and sensation-seeking among urban, low-income African-American adolescents. *Journal of the National Medical Association*, *93*(4), 129–138.

Strandberg, A., Skoglund, C., Gripenberg, J., & Kvillemo, P. (2019). Alcohol and illicit drug consumption and the association with risky sexual behaviour among Swedish youths visiting youth health clinics. *Nordic Studies on Alcohol and Drugs*, *36*(5), 442–459. https://doi.org/10.1177/1455072519845970

Tan, R. K. J., O’Hara, C. A., Koh, W. L., Le, D., Tan, A., Tyler, A., Tan, C., Kwok, C., Banerjee, S., & Wong, M. L. (2021). Social capital and chemsex initiation in young gay, bisexual, and other men who have sex with men: The pink carpet Y cohort study. *Substance Abuse: Treatment, Prevention, and Policy*, *16*(1), num. 18. <https://doi.org/10.1186/s13011-021-00353-2>

Thompson, R. G., Aivadyan, C., Stohl, M., Aharonovich, E., & Hasin, D. S. (2020). Smartphone application plus brief motivational intervention reduces substance use and sexual risk behaviors among homeless young adults: Results from a randomized controlled trial. *Psychology of Addictive Behaviors*, *34*(6), 641–649. https://doi.org/10.1037/adb0000570

Thompson, R. G., Eaton, N. R., Hu, M. C., Grant, B. F., & Hasin, D. S. (2014). Regularly drinking alcohol before sex in the United States: Effects of relationship status and alcohol use disorders. *Drug and Alcohol Dependence*, *141*, 167–170. <https://doi.org/10.1016/j.drugalcdep.2014.05.021>

Tolou-Shams, M., Brown, L. K., Gordon, G., & Fernandez, I. (2007). Arrest history as an indicator of adolescent/young adult substance use and HIV risk. *Drug and Alcohol Dependence*, *88*, 87–90. <https://doi.org/10.1016/j.drugalcdep.2006.09.017>

Tolou-Shams, M., Brown, L. K., Houck, C., & Lescano, C. M. (2008). The association between depressive symptoms, substance use, and HIV risk among youth with an arrest history. *Journal of Studies on Alcohol and Drugs*, *69*(1), 58–64. https://doi.org/10.15288/jsad.2008.69.58

Wechsberg, W. M., Luseno, W. K., Karg, R. S., Young, S., Rodman, N., Myers, B., & Parry, C. D. H. (2008). Alcohol, cannabis, and methamphetamine use and other risk behaviours among black and coloured South African women: A small randomized trial in the Western Cape. *International Journal of Drug Policy*, *19*(2), 130–139. https://doi.org/10.1016/j.drugpo.2007.11.018

Wells, B. E., Kelly, B. C., Golub, S. A., Grov, C., & Parsons, J. T. (2010). Patterns of alcohol consumption and sexual behavior among young adults in nightclubs. *American Journal of Drug and Alcohol Abuse*, *36*(1), 39–45. https://doi.org/10.3109/00952990903544836

Wesche, R., Lefkowitz, E. S., & Maggs, J. L. (2021). Short-term consequences of sex: Contextual predictors and change across college. *Archives of Sexual Behavior*, *50*(4), 1613–1626. https://doi.org/10.1007/s10508-020-01874-w

Wheater, C. P., Cook, P. A., Clark, P., Syed, Q., & Bellis, M. A. (2003). Re-emerging syphilis: A detrended correspondence analysis of the behaviour of HIV positive and negative gay men. *BMC Public Health*, *3*, 34. https://doi.org/10.1186/1471-2458-3-34

Wilton, L. (2008). Correlates of substance use in relation to sexual behavior in black gay and bisexual men: Implications for HIV prevention. *Journal of Black Psychology*, *34*(1), 70–93. https://doi.org/10.1177/0095798407310536

Young, L. E., Jonas, A. B., Michaels, S., Jackson, J. D., Pierce, M. L., & Schneider, J. A. (2017). Social-structural properties and HIV prevention among young men who have sex with men in the ballroom house and independent gay family communities. *Social Science and Medicine*, *174*, 26–34. https://doi.org/10.1016/j.socscimed.2016.12.009

Zullig, K. J., Young, M., & Hussain, M. (2010). Distinguishing between positive and negative social bonding in problem drinking among college students. *American Journal of Health Education*, *41*(2), 85–92. https://doi.org/10.1080/19325037.2010.10599132
