# Supplementary material for: Prevalence of Intoxicating Substance Use Before or During Sex Among Young Adults: A Systematic Review and Meta-Analysis
Source: Arch Sex Behav. 2023 Mar 10;52(6):2503–26. doi: 10.1007/s10508-023-02572-z (PMC10501956; doi:10.1007/s10508-023-02572-z)
Supplement: Supplementary file 3 — Supplementary file3 (DOCX 37 KB) [file 10508_2023_2572_MOESM3_ESM.docx]

**Supplementary Table S3**

*Characteristics of included studies*

| **Nº** | **Study** | **Doc** | **De** | **Sa** | **AP** | **Country** | **n** | **W (%)** | **Min age** | **Max age** | **Mean or Median** | **Global P.**  **(%)** | **Marijuana (%)** | **Alcohol (%)** | **Cocaine (%)** | **Heroin (%)** |
| --- | --- | --- | --- | --- | --- | --- | --- | --- | --- | --- | --- | --- | --- | --- | --- | --- |
| 1 | Anton Ruiz & Espada (2009) | A-PR | Cs | NP | Q | Spain | 339 | 63.13 | 18 | 29 | 19.53 | NA | 16.22 | 50.15 | 4.72 | NA |
| 2 | Apostolopoulos et al. (2002) | A-PR | Cs | QP | S | USA | 534 | 60.11 | 18 | 25 | NA | 44.19 | NA | 44.19 | NA | NA |
| 3 | Aung & Panza (2016) | A-PR | Cs | NP | MM | Thailand | 362 | 39.78 | 18 | 24 | 22.4 | 12.98 | NA | 12.98 | NA | NA |
| 4 | Baćak & Štulhofer (2012) | A-PR | Cs | QP | MM | Croatia | 679 | 45.95 | 18 | 25 | NA | NA | NA | 79.68 | NA | NA |
| 5 | Bianchi & Popper (2000) | A-PR | Cs | NP | Q | Slovakia | 432 | NA | NA | NA | 20.15 | 51.39 | NA | NA | NA | NA |
| 6 | Boone & Lefkowitz (2004) | A-PR | Cs | NP | S | USA | 154 | 62.34 | 18 | 25 | 20.8 | 70.13 | NA | 70.13 | NA | NA |
| 7 | Braithwaite et al. (2015) | A-PR | Cs | NP | S | USA | 1216 | 63.08 | 18 | 25 | 19.35 | 55.02 | NA | NA | NA | NA |
| 8 | Brown & Vanable (2007) | A-PR | Cs | NP | Q | USA | 330 | 66.97 | 18 | NA | 18.9 | 32.12 | NA | 32.12 | NA | NA |
| 9 | Brown (2000) | A-PR | Cs | NP | Q | USA | 407 | 69.04 | 18 | 21 | 19.,4 | NA | NA | 59.34 | NA | NA |
| 10 | Chirinda & Peltzer (2014) | A-PR | Cs | NP | Q | South Africa | 2138 | 47.19 | 18 | 24 | NA | NA | NA | 13.61 | NA | NA |
| 11 | Choi et al. (2005) | A-PR | Cs | NP | Q | USA | 496 | NA | 18 | 29 | 25 | NA | 13.91 | 32.26 | NA | 0 |
| 12 | Christian (2001) | T-NPR | Cs | NP | Q | USA | 106 | 100 | 18 | 25 | 20.03 | 28.30 | NA | NA | NA | NA |
| 13 | Cohan et al. (2005) | A-PR | Cs | QP | I | USA | 2543 | 100 | 18 | 29 | NA | NA | NA | 27.84 | 4.09 | 1.06 |
| 14 | D Anna et al. (2021) | A-PR | L | NP | Q | USA | 250 | NA | 18 | 25 | 22 | 37.90 | NA | NA | NA | NA |
| 15 | Des Rosiers et al. (2013) | A-PR | Cs | NP | S | USA | 1527 | 76.03 | 18 | 25 | 20.35 | 16.37 | NA | 16.37 | NA | NA |
| 16 | Dong et al. (2015) | A-PR | Cs | NP | I | China | 358 | 100 | 18 | 29 | 23 | 53.35 | NA | 53.35 | NA | NA |
| 17 | Fantasia et al. (2015) | A-PR | Cs | NP | S | USA | 925 | 100 | 18 | 25 | 20.6 | 59.03 | NA | 59.03 | NA | NA |
| 18 | Feinstein et al. (2018) | A-PR | RCT | NP | S | USA | 813 | NA | 18 | 29 | 24.27 | NA | 27.31 | NA | NA | NA |
| 19 | Guimaraes et al. (2014) | A-PR | Cs | NP | I | Brazil | 267 | 41.2 | 18 | 25 | NA | 34.08 | NA | NA | NA | NA |
| **Nº** | **Study** | **Doc** | **De** | **Sa** | **AP** | **Country** | **n** | **W (%)** | **Min age** | **Max age** | **Mean or Median** | **Global P.**  **(%)** | **Marijuana (%)** | **Alcohol (%)** | **Cocaine**  **(%)** | **Heroin (%)** |
| 20 | Hamilton et al. (2019) | A-PR | Cs | NP | S | USA | 4284 | 68.3 | 18 | 25 | 20.9 | 9.16 | NA | NA | NA | NA |
| 21 | Hoque & Ghuman (2011) | A-PR | Cs | P | S | South Africa | 752 | 52 | NA | NA | 21.57 | NA | NA | 16.36 | NA | NA |
| 22 | Jackson (2014) | T-NPR | Q-E | NP | S | USA | 118 | 82.20 | 18 | 20 | 19 | 46.61 | NA | 46.61 | NA | NA |
| 23 | Jones & Hoover (2018) | A-PR | CT | NP | I | USA | 238 | 100 | 18 | 29 | 22 | 78.57 | NA | NA | NA | NA |
| 24 | Jones (2001) | T-NPR | Cs | NP | I | USA | 257 | 100 | 18 | 29 | 21.62 | 40.23 | NA | NA | NA | NA |
| 25 | Kim et al. (2007) | A-PR | Cs | P | Q | USA | 815 | 55.70 | 18 | NA | 24 | 15.11 | NA | 15.11 | NA | NA |
| 26 | Kim et al. (2019) | A-PR | Cs | NP | S | USA | 320 | 75.93 | 18 | 25 | 20.26 | 62.81 | NA | NA | NA | NA |
| 27 | Kogan et al. (2015) | A-PR | Cs | NP | I | USA | 361 | NA | 19 | 22 | 20.3 | 61.5 | NA | NA | NA | NA |
| 28 | Loza et al. (2021) | A-PR | Cs | NP | S | Mexico | 95 | 53.68 | 18 | 24 | 22.6 | NA | 25.61 | 59.76 | 9.76 | 3.66 |
| 29 | Makgale & Plattner (2017) | A-PR | Cs | NP | Q | Botswana | 309 | 64.40 | 18 | 27 | 20.3 | NA | NA | 10.03 | NA | NA |
| 30 | Mayo-Wilson et al. (2020) | A-PR | Cs | NP | S | Republic of Kenya | 350 | 44 | 18 | 22 | 19.4 | 24.57 | NA | NA | NA | NA |
| 31 | Mcharo et al. (2020) | A-PR | Cs | P | Q | Tanzania | 504 | 43.05 | 18 | 24 | 21.5 | NA | NA | 5.56 | NA | NA |
| 32 | Merianos, King & Vidourek (2013) | A-PR | Cs | QP | Q | USA | 465 | 64.52 | 18 | NA | 21.62 | NA | NA | 59.57 | NA | NA |
| 33 | Metzger (2015) | T-NPR | Cs | NP | S | USA | 228 | 74.12 | 18 | 25 | 20.53 | 13.16 | NA | 13.16 | NA | NA |
| 34 | Meuwly et al. (2021) | A-PR | Cs | P | Q | Switzerland | 3892 | 47.79 | 24 | 26 | 26.35 | 49.59 | NA | NA | NA | NA |
| 35 | Miller et al. (2004) | A-PR | Cs | NP | Q | USA | 481 | 49.69 | NA | NA | 21 | 52 | NA | NA | NA | NA |
| 36 | Neuman et al. (2019) | A-PR | Cs | NP | S | USA | 15 | 53.33 | 18 | 24 | NA | 40 | NA | NA | NA | NA |
| 37 | Otiniano et al. (2020) | A-PR | Cs | NP | MM | USA | 356 | 42.42 | 18 | 25 | 20.9 | NA | 43.26 | 35.67 | NA | NA |
| 38 | Palamar et al. (2018) | A-PR | Cs | NP | I | USA | 679 | 38.73 | 18 | 25 | 21.89 | NA | 50.81 | 61.86 | NA | NA |
| 39 | Peterson (2013) | T-NPR | Q-E | NP | Q | USA | 193 | 66.84 | 18 | NA | 19.33 | 54.92 | NA | 54.92 | NA | NA |
| 40 | Powell (2018) | T-NPR | L | NP | S | USA | 630 | 71.11 | 18 | 24 | NA | 53.57 | NA | NA | NA | NA |
| 41 | Reid et al. (2008) | A-PR | Ch | NP | Q | Canada | 192 | NA | 19 | 20 | 19.49 | 8.33 | NA | NA | NA | NA |
| 42 | Ristuccia et al. (2018) | A-PR | Ch | NP | I | USA | 500 | NA | 22 | 23 | NA | 45 | NA | NA | NA | NA |
| 43 | Rizwan et al. (2014) | A-PR | Cs | NP | I | India | 162 | NA | 18 | NA | 21.4 | 48.15 | NA | 48.15 | NA | NA |
| 44 | Roberts & Kennedy (2006) | A-PR | Cs | NP | Q | USA | 100 | 100 | 18 | 24 | 20.2 | 52 | NA | NA | NA | NA |

| **Nº** | **Study** | **Doc** | **De** | **Sa** | **AP** | **Country** | **n** | **W (%)** | **Min age** | **Max age** | **Mean or Median** | **Global P.**  **(%)** | **Marijuana (%)** | **Alcohol (%)** | **Cocaine (%)** | **Heroin (%)** |
| --- | --- | --- | --- | --- | --- | --- | --- | --- | --- | --- | --- | --- | --- | --- | --- | --- |
| 45 | Saengdidtha et al. (2016) clinic | A-PR | Cc | NP | Q | Thailand | 203 | NA | 21 | NA | 21.9 | NA | NA | 67 | NA | NA |
| 46 | Saengdidtha et al. (2016) non-clinic | A-PR | Cc | NP | Q | Thailand | 234 | NA | 21 | NA | 21.9 | NA | NA | 53.85 | NA | NA |
| 47 | Santos et al. (2018) | A-PR | Cs | QP | S | Portugal | 1946 | 36.02 | 18 | 29 | 20.7 | NA | NA | 33 | NA | NA |
| 48 | Sawyer et al. (2018) | A-PR | Cs | NP | S | USA | 509 | 71.31 | 18 | 25 | 20.08 | NA | NA | 34.38 | NA | NA |
| 49 | Schwartz et al. (2011) | A-PR | Cs | NP | S | USA | 9515 | 73 | 18 | 25 | 19.75 | 27.4 | NA | NA | NA | NA |
| 50 | Scott-Sheldon et al. (2008) | A-PR | Cs | NP | Q | USA | 1595 | 64.01 | NA | NA | 19.,5 | 8.03 | NA | NA | NA | NA |
| 51 | Snipes & Benotsch (2013) | A-PR | Cs | NP | S | USA | 704 | 59.94 | 18 | NA | 19.01 | NA | NA | 32.53 | NA | NA |
| 52 | So et al. (2005) | A-PR | Cs | NP | Q | USA | 248 | 54.03 | 18 | 27 | 20 | NA | NA | 6.05 | NA | NA |
| 53 | Tan et al. (2021) | A-PR | Ch | NP | S | Republic of Singapore | 570 | NA | 18 | 25 | 21.7 | NA | NA | 33.33 | NA | NA |
| 54 | Vail-Smith et al. (2010) fall sample | A-PR | Cs | NP | S | USA | 905 | 67.73 | 18 | 20 | 18.1 | 22.32 | NA | NA | NA | NA |
| 55 | Vail-Smith et al. (2010) spring sample | A-PR | Cs | NP | S | USA | 904 | 69.58 | 18 | 20 | 18.6 | 27.43 | NA | NA | NA | NA |
| 56 | Villegas Pantoja et al. (2021) | A-PR | Cs | QP | Q | Mexico | 304 | 100 | 18 | 24 | 20.42 | 29.93 | NA | 29.93 | NA | NA |
| 57 | Walsh et al. (2020) | A-PR | Cs | NP | S | USA | 336 | 74.40 | 18 | 29 | 20.5 | 29.17 | NA | 29.17 | NA | NA |

*Table legend*: NA = Not applicable; Do = Document type; De = Design; Sa = Sampling; AP = Administration Procedure; Global P. = Global Prevalence; A-PR = Article – Peer Reviewed; T-NPR = Thesis - Not Peer Reviewed; Cs = Cross-sectional; L = Longitudinal; Q-E = Quasi-experimental trial; RCT = Randomized Control Trial (Baseline); Cc = Case control study; Ch = Cohort study; P = Probabilistic; NP = Non-probabilistic; QP = Quasi-Probabilistic; Q = Questionnaire; S = Survey; I = Interview; MM = Mixed Methods
