# Supplementary material for: Prevalence of Intoxicating Substance Use Before or During Sex Among Young Adults: A Systematic Review and Meta-Analysis
Source: Arch Sex Behav. 2023 Mar 10;52(6):2503–26. doi: 10.1007/s10508-023-02572-z (PMC10501956; doi:10.1007/s10508-023-02572-z)
Supplement: Supplementary file 4 — Supplementary file4 (DOCX 32 KB) [file 10508_2023_2572_MOESM4_ESM.docx]

**Supplementary Table S4**

*Critical appraisal of the studies*

| **Nº** | **Study** | **Population** | **Full age description** | **Study design** | **Sampling technique** | **Instrument/measure quality: reported or justified validity and/or reliability in the sample or comparable samples** | **Common administration procedure or measure for all participants** | **Timeframe** | **RR** | **Risk of bias** |
| --- | --- | --- | --- | --- | --- | --- | --- | --- | --- | --- |
| 1 | Anton Ruiz and Espada (2009) | University students | Yes | Cross-sectional | Non-probabilistic | Not reported | Yes | Lifetime or not defined | NA | High |
| 2 | Apostolopoulos et al. (2002) | Undergraduate students | Yes | Cross-sectional | Quasi-probabilistic | Not reported | Yes | Last spring vacation | NA | Moderate |
| 3 | Aung and Panza (2016) | Young Myanmar migrant workers | Yes | Cross-sectional | Non-probabilistic | Not reported | Yes | Not defined | NA | High |
| 4 | Baćak and Štulhofer (2012) | Young Croatian adults | Yes | Cross-sectional | Quasi-probabilistic | Reported or justified validity/reliability | Yes | Last year | NA | Low |
| 5 | Bianchi and Popper (2000) | Slovak soldiers | Incomplete | Cross-sectional | Non-probabilistic | Not reported | Yes | Last casual sex | NA | High |
| 6 | Boone and Lefkowitz (2004) | Late adolescent college students | Yes | Cross-sectional | Non-probabilistic | Not reported | Yes | Lifetime or not defined | NA | High |
| 7 | Braithwaite et al. (2015) | Undergraduate students | Yes | Cross-sectional | Non-probabilistic | Not reported | Yes | Last twelve months | NA | High |
| 8 | Brown (2000) | Young College Students | Yes | Cross-sectional | Non-probabilistic | Reported or justified validity/reliability | Yes | Last year | 45 to 90% | Moderate |
| 9 | Brown and Vanable (2007) | College students | Incomplete | Cross-sectional | Non-probabilistic | Not reported | Yes | Last sexual intercourse | NA | High |
| 10 | Chirinda and Peltzer (2014) | Youth | Yes | Cross-sectional | Non-probabilistic | Not reported | Yes | Last three months | 96.40% | High |
| 11 | Choi et al. (2005) | Young Asian and Pacific Islander MSM | Yes | Cross-sectional | Non-probabilistic | Not reported | Yes | Last six months | NA | High |
| 13 | Christian (2001) | African American Women | Yes | Cross-sectional | Non-probabilistic | Not reported | Yes | Last year | NA | High |
| 12 | Cohan et al. (2005) | Low income women | Yes | Cross-sectional | Quasi-probabilistic | Not reported | Yes | Not defined | 71.50% | Moderate |
| 14 | D’Anna et al. (2021) | Young Black MSM | Yes | Longitudinal | Non-probabilistic | Not reported | Tablet or paper format | Last three months | NA | High |
| 15 | Des Rosiers et al. (2013) | Hispanic College Students | Yes | Cross-sectional | Non-probabilistic | Not reported | Yes | Last month | NA | High |
| 16 | Dong et al. (2015) | Young female migrants | Yes | Cross-sectional | Non-probabilistic | Not reported | Yes | Last six months | NA | High |

| **Nº** | **Study** | **Population** | **Full age description** | **Study design** | **Sampling technique** | **Instrument/measure quality: reported or justified validity and/or reliability in the sample or comparable samples** | **Common administration procedure or measure for all participants** | **Timeframe** | **RR** | **Risk of bias** |
| --- | --- | --- | --- | --- | --- | --- | --- | --- | --- | --- |
| 17 | Fantasia et al. (2015) | College women | Yes | Cross-sectional | Non-probabilistic | Reported or justified validity/reliability | Yes | Lifetime or not defined | NA | Moderate |
| 18 | Feinstein et al. (2018) | Young MSM | Yes | Randomized control trial | Non-probabilistic | Not reported | Yes | Last three months | NA | High |
| 19 | Guimaraes et al. (2014) | Young adults | Yes | Cross-sectional | Non-probabilistic | Reported or justified validity / reliability | Yes | Lifetime or not defined | NA | Moderate |
| 20 | Hamilton et al. (2019) | College students | Yes | Cross-sectional | Non-probabilistic | Not reported | Yes | Lifetime or not defined | 28.70% | High |
| 21 | Hoque and Ghuman (2011) | Undergraduate students | Incomplete | Cross-sectional | Probabilistic | Not reported | Yes | Not defined | 99% | Moderate |
| 22 | Jackson (2014) | College students | Yes | Quasi-experimental trial | Non-probabilistic | Not reported | Yes | Currently | NA | High |
| 23 | Jones (2001) | Young urban women | Yes | Cross-sectional | Non-probabilistic | Not reported | Yes | Last three months | NA | High |
| 24 | Jones and Hoover (2018) | Young urban women | Yes | Clinic trial | Non-probabilistic | Not reported | Yes | Last three months | NA | High |
| 25 | Kim et al. (2007) | University students | Incomplete | Cross-sectional | Probabilistic | Reported or justified validity/reliability | Yes | Last year | 80.05% | Low |
| 26 | Kim et al. (2019) | Undergraduate students | Yes | Cross-sectional | Non-probabilistic | Reported or justified validity/reliability | Yes | Lifetime or not defined | NA | Moderate |
| 27 | Kogan et al. (2015) | Young rural african american men | Yes | Cross-sectional | Non-probabilistic | Not reported | Yes | Last three months | NA | High |
| 28 | Loza et al. (2021) | University and Community Young Adults | Yes | Cross-sectional | Non-probabilistic | Unclear | Yes | Lifetime or not defined | NA | High |
| 29 | Makgale and Plattner (2017) | Undergraduate students | Yes | Cross-sectional | Non-probabilistic | Reported or justified validity/reliability | Yes | Last three months | NA | High |
| 30 | Mayo-Wilson et al. (2020) | Young adults | Yes | Cross-sectional | Non-probabilistic | Not reported | Yes | Last six months | NA | High |
| 31 | Mcharo et al. (2020) | Students of Higher Learning Institutions | Yes | Cross-sectional | Probabilistic | Not reported | Tablet or smartphone, or hard copy | Lifetime or not defined | 79.74% | Moderate |
| 32 | Merianos et al. (2013) | University students | Incomplete | Cross-sectional | Quasi-probabilistic | Reported or justified validity/reliability | Yes | Lifetime or not defined | 93% | Low |
| 33 | Metzger (2015) | African American students | Yes | Cross-sectional | Non-probabilistic | Justified | Yes | Last sexual intercourse | NA | Moderate |

| Nº | **Study** | **Population** | **Full age description** | **Study design** | **Sampling technique** | **Instrument/measure quality: reported or justified validity and/or reliability in the sample or comparable samples** | **Common administration procedure or measure for all participants** | **Timeframe** | **RR** | **Risk of bias** |
| --- | --- | --- | --- | --- | --- | --- | --- | --- | --- | --- |
| 34 | Meuwly et al. (2021) | Heterosexual young adults | Yes | Cross-sectional | Probabilistic | Not reported | Yes | Lifetime or not defined | 15.10% | Moderate |
| 35 | Miller et al. (2004) | Middle school students | Incomplete | Cross-sectional | Non-probabilistic | Not reported | Yes | Last three months | NA | High |
| 36 | Neuman et al. (2019) | College students | Yes | Cross-sectional | Non-probabilistic | Reported or justified validity / reliability | Yes | Last sexual intercourse | 25% | Moderate |
| 37 | Otiniano et al. (2020) | Caribbean Latinx young adults | Yes | Cross-sectional | Non-probabilistic | Not reported | Yes | Last year | 73% | High |
| 38 | Palamar et al. (2018) | Young people attending electronic dance music venues | Yes | Cross-sectional | Non-probabilistic | Not reported | Yes | Lifetime or not defined | 63% | High |
| 39 | Peterson (2013) | College students | Incomplete | Quasi-experimental | Non-probabilistic | Not reported | Yes | Last six months | NA | High |
| 40 | Powell (2018) | Undergraduate students | Yes | Longitudinal | Non-probabilistic | Unclear | Yes | Last year | NA | High |
| 41 | Reid et al. (2008) | Young adults with congenital heart disease | Yes | Cohort study | Non-probabilistic | Reported or justified validity/reliability | Yes | Last month | 63% | Moderate |
| 42 | Ristuccia et al. (2018) | Sexual Minority Young Adults | Yes | Cohort study | Non-probabilistic | Not reported | Yes | Last month | NA | High |
| 43 | Rizwan et al. (2014) | Male migrant workers | Incomplete | Cross-sectional | Non-probabilistic | Not reported | Yes | Last non-spousal sexual encounter | NA | High |
| 44 | Roberts and Kennedy (2006) | Young multiethnic college women | Yes | Cross-sectional | Non-probabilistic | Not reported | Yes | Last four sexual episodes | NA | High |
| 45 | Saengdidtha et al. (2016) | Thai army constricts young men | Incomplete | Case control study | Non-probabilistic | Not reported | Yes | Lifetime or not defined | NA | High |
| 46 | Santos et al. (2018) | University students | Yes | Cross-sectional | Quasi-probabilistic | Not reported | Yes | Last twelve months | 98.30% | Moderate |
| 47 | Sawyer et al. (2018) | Young adults | Yes | Cross-sectional | Non-probabilistic | Reported or justified validity/reliability | Yes | Last three months | NA | Moderate |
| 48 | Schwartz et al. (2011) | Undergraduate students | Yes | Cross-sectional | Non-probabilistic | Not reported | Yes | Last month | NA | High |
| 49 | Scott-Sheldon et al. (2008) | Undergraduate students | Incomplete | Cross-sectional | Non-probabilistic | Not reported | Yes | Last three months | NA | High |
| 50 | Snipes and Benotsch (2013) | Undergraduate students | Incomplete | Cross-sectional | Non-probabilistic | Justified | Yes | Last three months | NA | Moderate |
| 51 | So et al. (2005) | Asian American College Students | Yes | Cross-sectional | Non-probabilistic | Not reported | Yes | Last month | NA | High |
| **Nº** | **Study** | **Population** | **Full age description** | **Study design** | **Sampling technique** | **Instrument/measure quality: reported or justified validity and/or reliability in the sample or comparable samples** | **Common administration procedure or measure for all participants** | **Timeframe** | **RR** | **Risk of bias** |
| 52 | Tan et al. (2021) | Young MSM | Yes | Cohort study | Non-probabilistic | Not reported | Yes | Lifetime or not defined | NA | High |
| 53 | Vail-Smith et al. (2010) | Freshmen or First-year students | Yes | Cross-sectional | Non-probabilistic | Reported or justified validity/reliability | Yes | Last sexual intercourse | 67.3% and 84% | Moderate |
| 54 | Villegas Pantoja et al. (2021) | University women students | Yes | Cross-sectional | Quasi-probabilistic | Not reported | Yes | Lifetime or not defined | NA | Moderate |
| 55 | Walsh et al. (2020) | Undergraduate students | Yes | Cross-sectional | Probabilistic | Not reported | Yes | Last twelve months | 63.68% | Moderate |

*Table legend*: NA = Not applicable; RR= Response Rate.
